# Characterization of the zinc finger μ-protein HVO_0758 from *Haloferax volcanii*: biological roles, zinc binding, and NMR solution structure

**DOI:** 10.3389/fmicb.2023.1280972

**Published:** 2023-11-29

**Authors:** Deniz Üresin, Dennis J. Pyper, Andreas Borst, Lydia Hadjeras, Rick Gelhausen, Rolf Backofen, Cynthia Sharma, Harald Schwalbe, Jörg Soppa

**Affiliations:** ^1^Institute for Molecular Biosciences, Goethe University, Frankfurt, Germany; ^2^Institute for Organic Chemistry and Chemical Biology, Center for Biomolecular Magnetic Resonance (BMRZ), Goethe University, Frankfurt, Germany; ^3^Institute of Molecular Infection Biology, University of Würzburg, Würzburg, Germany; ^4^Bioinformatics Group, Department of Computer Science, University of Freiburg, Freiburg, Germany; ^5^Signalling Research Centres BIOSS and CIBSS, University of Freiburg, Freiburg, Germany

**Keywords:** Archaea, *Haloferax volcanii*, small proteins, microproteins, zinc finger, TALON, NMR solution structure, RNA-Seq

## Abstract

It is increasingly recognized that very small proteins (μ-proteins) are ubiquitously found in all species of the three domains of life, and that they fulfill important functions. The halophilic archaeon *Haloferax volcanii* contains 282 μ-proteins of less than 70 amino acids. Notably, 43 of these contain two C(P)XCG motifs, suggesting their potential to complex a zinc ion. To explore the significance of these proteins, 16 genes encoding C(P)XCG proteins had been deleted, and the majority of mutants exhibited phenotypic differences to the wild-type. One such protein, HVO_2753, was thoroughly characterized in a previous study. In the present study an in-depth analysis of a second protein, HVO_0758, was performed. To achieve this goal, the HVO_0758 protein was produced heterologously in *Escherichia coli* and homologously in *H. volcanii*. The purified protein was characterized using various biochemical approaches and NMR spectroscopy. The findings demonstrated that HVO_0758 is indeed a *bona fide* zinc finger protein, and that all four cysteine residues are essential for folding. The NMR solution structure was solved, revealing that HVO_0758 is comprised of an N-terminal alpha helix containing several positively charged residues and a globular core with the zinc finger domain. The transcriptomes of the *HVO_0758* deletion mutant and, for comparison, the *HVO_2753* deletion mutant were analyzed with RNA-Seq and compared against that of the wild-type. In both mutants many motility and chemotaxis genes were down-regulated, in agreement to the phenotype of the deletion mutants, which had a swarming deficit. The two *H. volcanii* zinc-finger μ-proteins HVO_0758 and HVO_2753 showed many differences. Taken together, two zinc finger μ-proteins of *H. volcanii* have been characterized intensively, which emerged as pivotal contributors to swarming behavior and biofilm formation.

## Introduction

Until quite recently, small proteins have been overlooked for several reasons. In genome sequencing projects, open reading frames (ORFs) comprising fewer than 100 codons were by default not recognized as potential genes. This was a deliberate strategy to prevent the annotation of a large number of false positive gene predictions. Additionally, very small proteins disappeared in experimental approaches that were optimized for the characterization of normal-sized and large proteins. Furthermore, biochemical characterization of very small proteins faces specific challenges, for example because they often do not exhibit a specific fold in isolated form (Kubatova et al., 2020). Nevertheless, the past few years have unveiled the existence and vital roles of very small proteins across all three domains of life. Notable advancements encompass the establishment of ribosome profiling methods, advances in mass spectrometry (peptidomics), and enhancements in gene/ORF prediction through bioinformatics refinements. Several recent reviews summarize current knowledge about very small proteins in archaea, bacteria, and eukaryotes (Storz et al., 2014; Orr et al., 2020; Cassidy et al., 2021; Steinberg and Koch, 2021; Tharakan and Sawa, 2021; Chen et al., 2022; Gray et al., 2022; Leong et al., 2022; Schlesinger and Elsässer, 2022; Weidenbach et al., 2022; Zhang et al., 2022; Dong et al., 2023; Hassel et al., 2023).

In contrast to the many novel findings the field is so new that a common nomenclature does not exist. Very small proteins are denoted as μ-peptides, peptides, μ-proteins, small proteins, and sORF encoded proteins. In addition, the upper size limit varies from about 50 aa to 100 aa. In this contribution the name “μ-proteins” will be used, to distinguish these proteins from “small proteins,” which are much smaller than an average sized protein of 300 aa (prokaryotes), but larger than the annotation limit of 100 aa.

Even before the general importance of μ-proteins in all domains of life was recognized, the group of the late Dieter Oesterhelt characterized the “low molecular weight proteome” of the halophilic archaeon *Halobacterium salinarum* (Klein et al., 2007). They identified 380 proteins smaller than 20 kDa, constituting around 9% of the proteome, mostly lacking known functions. Approximately 20 of these proteins contained dual C(P)XCG motifs, suggesting a potential role in binding zinc ions, making them putative single-domain zinc finger proteins.

Zinc finger proteins were first discovered in eukaryotes and were thought for long to be confined to this domain (Maret, 2013). Eukaryotic zinc finger proteins are typically larger proteins housing multiple zinc finger domains. Zinc finger domains are characterized by four amino acids that complex a central zinc ion, e.g., four cysteines or two cysteines and two histidines. The four amino acids are organized into two motifs, CXXC or HXXH (X representing any amino acid), which are separated by a linker of variable length. C4 zinc fingers often display the more elaborate C(P)XCG consensus sequence (P in only one motif).

Zinc fingers are very versatile interaction domains, enabling zinc finger proteins to interact with diverse biomolecules, encompassing DNA, RNA, soluble and membrane proteins (Matthews and Sunde, 2002; Eom et al., 2016). Moreover, engineered zinc finger nucleases have been applied for gene editing (Bak et al., 2018). Structurally, zinc finger proteins have been classified into eight groups (Krishna et al., 2003).

Zinc finger proteins are not confined to eukaryotes, but are also present in prokaryotes. And, as noted above, not all zinc fingers are part of large proteins, small single domain zinc finger proteins also exist. In archaea, zinc finger proteins constitute 8% of small proteins with fewer than 100 amino acids, compared to a mere 1.5% in bacterial small proteins (Tarasov et al., 2008). In a recent study we have generated *in-frame* deletion mutants of 16 genes encoding zinc finger μ-proteins in the haloarchaeaon *Haloferax volcanii* (Nagel et al., 2019). 12 of these mutants exhibited distinct phenotypes from the wild-type, illustrating the diverse roles zinc finger μ-proteins play in haloarchaeal biological processes. These mutants did not only show loss-of-function characteristics, but also gain-of-function phenotypes, including improved biofilm formation (Nagel et al., 2019).

One protein, HVO_2753, was characterized in detail, including solving the NMR solution structure (Zahn et al., 2021). HVO_2753 was chosen because it is the only *H. volcanii* zinc finger protein with four C(P)XCG motifs, which are indicative for the formation of two zinc fingers. Surprisingly, a biochemical zinc assay and the NMR zinc titration and structure revealed consistently that HVO_2753 binds only one zinc ion. The second “zinc finger” has apparently lost the ability to bind a zinc ion, because the four cysteines do not point to the center of the “zinc finger.” This result shows that the presence of two C(P)XCG motifs in a protein sequence is highly indicative for the formation of a zinc finger and zinc binding, however, that exceptions exist and experimental verification is important.

Here, we report the detailed characterization of a second *H. volcanii* zinc finger μ-protein, HVO_0758. The protein was selected as its cognate deletion mutant showed interesting pleiotrophic phenotypes, i.e., a late onset of growth in synthetic glycerol medium, a lack of swarming, and enhanced biofilm formation. HVO_0758 was produced homologously in *H. volcanii* and heterologously in *Escherichia coli*, purified, and subjected to comprehensive characterization. Several conserved amino acids were selected and point mutants were generated. Notably, the NMR solution structure was solved with high resolution. Transcriptome comparisons were made between the deletion mutant, the wild-type, and the HVO_2753 deletion mutant. Lastly, characteristic features and the structures of the two in depth characterized proteins HVO_0758 and HVO_2753 were compared.

## Results

### Characteristic features of HVO_0758 and evolutionary conservation

**Figure 1:**
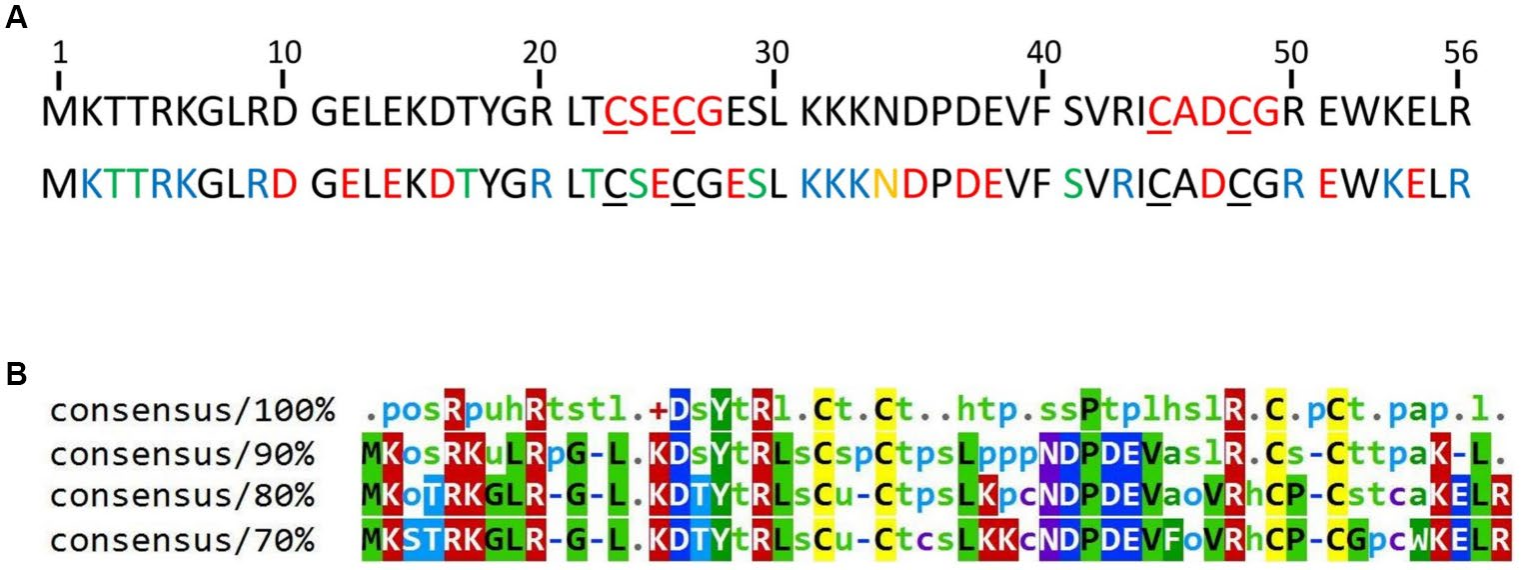
**(A)** Sequence of the protein HVO_0758. The four cysteines forming the zinc binding pocket are underlined. Top: The C(P)XCG motifs are highlighted in red. Bottom: Acidic amino acids are highlighted in red, basic residues in blue, residues with amide side chains in yellow and residues with hydroxyl groups in green. The accession number of the protein in the UniProtKB database is D4GTQ1. **(B)** Conservation of HVO_0758. A multiple sequence alignment was generated between HVO_0758 and the top 99 hits of a protein blast search. At each position the residues are shown that are, respectively, 100, 90, 80%, or 70% conserved. Lower-case letter legend: a → aromatic, h → hydrophobic, l → aliphatic, o → alcohol, p → polar, s → small (A, C, D, G, N, P, S, T, V), t → turnlike, u → tiny (A, G, S).

HVO_0758 was selected because a deletion mutant of the cognate gene exhibited a swarming deficit and a gain of function in biofilm formation, indicating that the protein fulfills important functions (Nagel et al., 2019). HVO_0758 is a protein of 56 amino acids with two C(P)XCG-like motifs, none of which contains a proline at the second position (Figure 1A). It is comprised of an extremely high fraction of charged and hydrophilic amino acids, and thus can be predicted to be involved in many interactions with other biomolecules. Notably, this amino acid composition includes 13 positively charged amino acids (lysine and arginine), and consequently HVO_0758 has an isoelectric point of 7.6. Such alleviated pK_i_ is very untypical for haloarchaeal proteins, which typically have a high fraction of negatively charged amino acids and a very low isoelectric point (Shukla, 2006; Oren, 2013). HVO_0758 is present in more than 200 other species of haloarchaea. However, there are no orthologs in other groups of archaea, in bacteria, or in eukaryotes. A multiple sequence alignment of 100 HVO_0758 orthologs revealed that many positions are highly conserved, in addition to the four cysteines (Figure 1B). Analysis of previous RNA-Seq and dRNA-Seq results (Babski et al., 2016; Laass et al., 2019) revealed that *HVO_0758* is expressed during exponential growth under optimal conditions, that the transcript is leaderless and that it has a long 3’-UTR of about 200 nt (Supplementary Figure S1). A Northern blot analysis confirmed that the transcript is considerably longer than the ORF and has a long UTR (see below).

### Heterologous production and characterization

In order to analyze the protein with NMR, it was heterologously produced in *E. coli* BL21(DE3) cells. The production was carried out in M9-medium with ^15^N-NH_4_Cl for single-labeled samples or ^15^N-NH_4_Cl and ^13^C-glucose for double-labeled samples as sole nitrogen and carbon sources. The protein was produced with an N-terminal His_6_-tag and a SUMO tag (Malakhov et al., 2004; Marblestone et al., 2006), which enabled the purification by twin-affinity chromatography. The tags were removed by the SUMO protease. Size exclusion chromatography was used as final purification step, resulting in a purity of >95%. The purification steps are visualized in Supplementary Figure S2.

**Figure 2:**
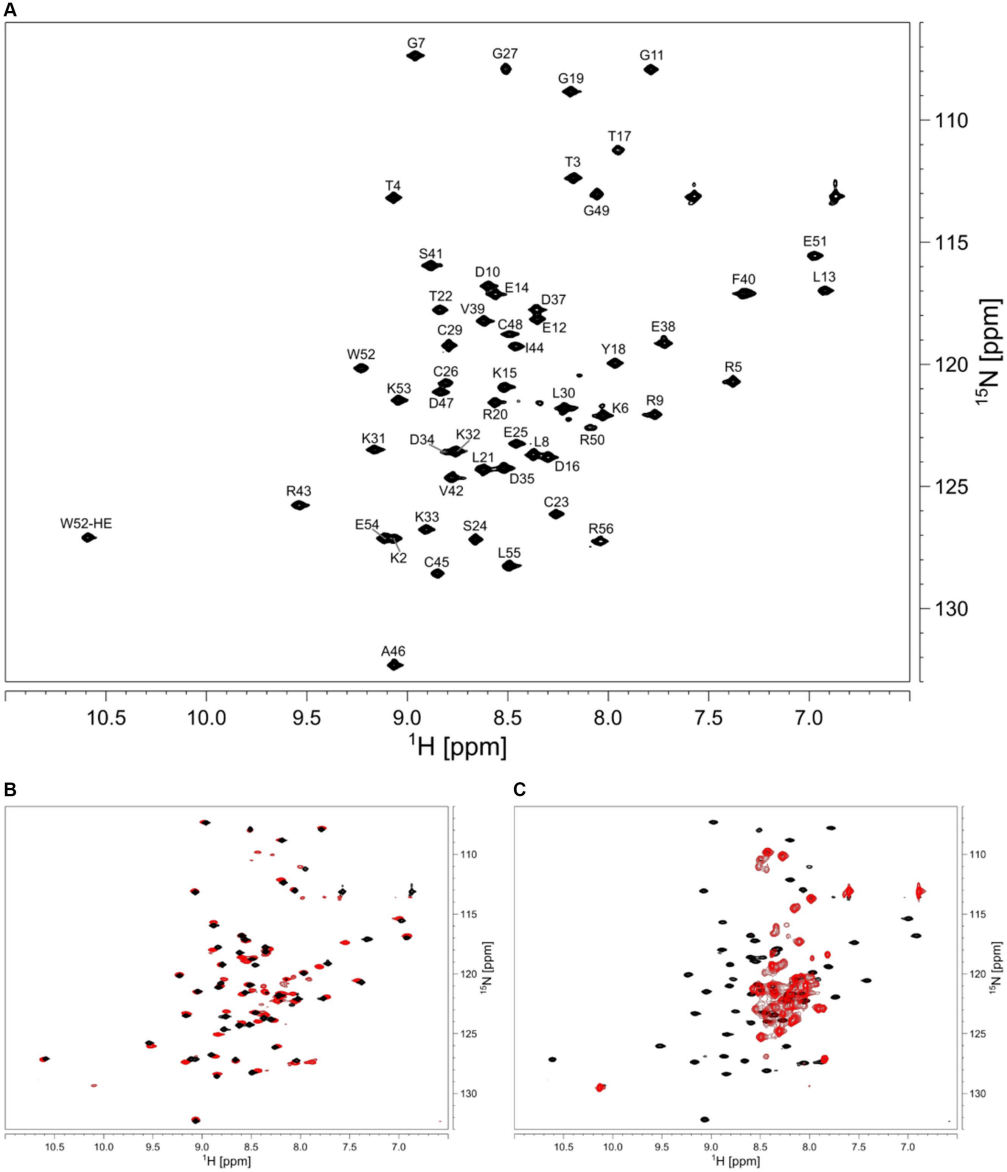
**(A)** 2D ^1^H ^15^N HSQC of HVO_0758. Assignment is shown next to the peaks. The sample contained 25 mM BisTris at pH 7, 1 M NaCl, 5 mM 2-mercaptoethanol, 5% D_2_O, 1 mM DSS. **(B)** Comparison of 2D ^1^H ^15^N HSQC spectra with different salt concentrations in the buffers. The sample for the black spectrum contained 1 M NaCl, the sample for the red spectrum contained 300 mM NaCl. **(C)** The red spectrum shows the effect of 10 mM EDTA added to the buffer, resulting in zinc-free, unfolded protein.

We aimed to characterize the effect of salt on the structure of HVO_0758 from the halophilic archaeon *H. volcanii,* which has an optimal salt concentration of 2.1 M NaCl. We started out with a NaCl concentration of 300 mM. The HSQC spectrum of HVO_0758 has 68 visible signals, although from the sequence only 54 signals are expected (Figure 2A). With 2D ^15^N-ZZ-exchange experiments (Montelione and Wagner, 1989; Farrow et al., 1994) we could show that these signals stem from two conformations that interconvert slowly, with one of them exhibiting a wide signal dispersion and the other a narrow, indicating that the protein is present in a structured and an unstructured conformation. This is further supported by the temperature series (Supplementary Figures S3, S4). At high temperatures (328 K) the protein completely unfolds and only the signals of the second conformation remain.

We increased the salt concentration of the protein buffer to 1 M NaCl, which led to the second (unstructured) conformation disappearing, showing that the folding of the protein is dependent on salt concentration (Figure 2B). Subsequent experiments for the investigation of structure and dynamics were thus carried out at 1 M NaCl.

### Zinc binding

In order to analyze the effects of zinc binding, all zinc ions were removed from the isolated protein by addition of 10 eq. of EDTA (Figure 2C). Without zinc ions, only signals of the unfolded conformation remained, indicating that zinc ions are necessary for the native folded structure. Addition of zinc ions to the unfolded protein fully refolded the protein.

### NMR assignment and TALOS prediction

**Figure 3:**
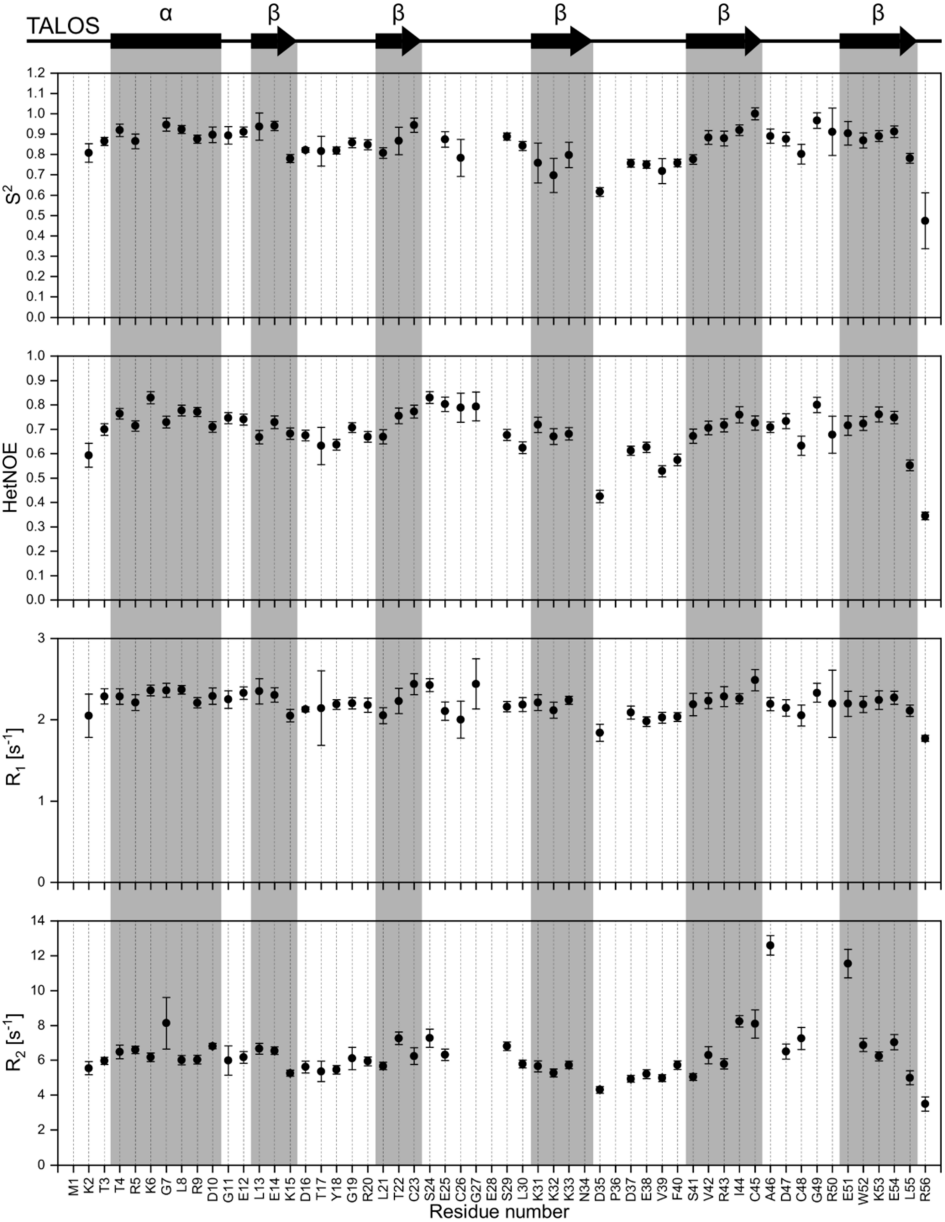
Dynamic studies of HVO_0758. On the top, the secondary structure prediction obtained from TALOS-N is shown. Below, the S^2^ order parameter (calculated with TENSOR2) is shown, which is based on the experimental relaxation parameters {^1^H^15^N}-hetNOE, R_1_ and R_2_.

NMR backbone and side-chain assignment were conducted manually using standard 2D and 3D experiments. The protein was measured with 1 M NaCl for correct folding conditions at 298 K. We were able to assign 99% of the backbone with the only missing signal being glutamate 28%. The assigned ^1^H-^15^N HSQC is shown in Figure 2A. With the backbone chemical shift assignments, we used TALOS-N (Shen and Bax, 2013) to predict secondary structure elements of the protein. We identified one α-helix ranging from T4 to D10, as well as five β-strands (L13 to K15, L21 to C23, K31 to N34, S41 to C45 and E51 to L55; Figure 3, top panel). Furthermore, we obtained backbone torsion angles from TALOS-N, which were used as constraints in the structure calculation (see below).

### Dynamic studies

We investigated the dynamic properties of HVO_0758 by analyzing T_1_ and T_2_ relaxation times as well as {^1^H^15^N}-hetNOE. The relaxation data were used to calculate the Lipari-Szabo order parameter S^2^, providing information about the local flexibility of the protein. Furthermore, we determined the experimental rotational correlation time τ_C_ to be 4.01 ns (Figure 3).

A temperature series ranging from 278 K to 333 K of ^1^H^15^N-HSQC spectra was recorded. From this, we monitored the temperature-dependent amide proton chemical shift perturbations. The extracted temperature coefficients provide information on secondary structure elements. Temperature coefficients below −4.5 ppb/K indicate weak hydrogen bonding, while temperature coefficients above −4.5 ppb/K indicate stronger hydrogen bonding and thus a more likely occurrence of secondary structures (Supplementary Figure S5).

Furthermore, we obtained ^3^J_HNHα_ coupling constants from a 3D HNHA spectrum which, based on the Karplus equation, can also be used to predict secondary structure formation (Supplementary Figure S6; Vuister and Bax, 1993). Coupling constants in the range of 3 to 4.5 Hz and 8 to 9 Hz indicate α-helices and β-sheets, respectively.

### 3D structure calculation

For the structure calculation, a total of 88 backbone torsion angles (φ, ψ) were obtained from TALOS-N and included in the structure calculation as restraints. In addition, 41 ^3^J_HNHα_ scalar couplings from a 3D HNHA spectrum were used and 12 hydrogen bonds were defined by manually checking visible cross-peaks in the 3D NOESY-HSQC spectra for potential secondary structure elements (Table 1).

**Table 1:** List of NOE values.

NOE distances	666
Short range (│i-j│ ≤ 1)	369
Medium range (1 < │i-j│ < 5)	73
Long range (│i-j│ ≥ 5)	224
Hydrogen bonds	12
Backbone torsion angles (φ,ψ)	88
Scalar couplings (^3^J(HN,Hα))	41
Lower length of S-S bond in zinc binding pocket	3.65 Å
Upper length of S-S bond in zinc binding pocket	4 Å
Number of S-S restraints in zinc binding pocket	6
Ramachandran score (%)	
Most favored	92.3
Additionally allowed	7.7
Generously allowed	0
Disallowed	0
Average rmsd to mean (Å)	
Backbone	0.36
All atoms	1.06
Restraint violations	0

**Figure 4:**
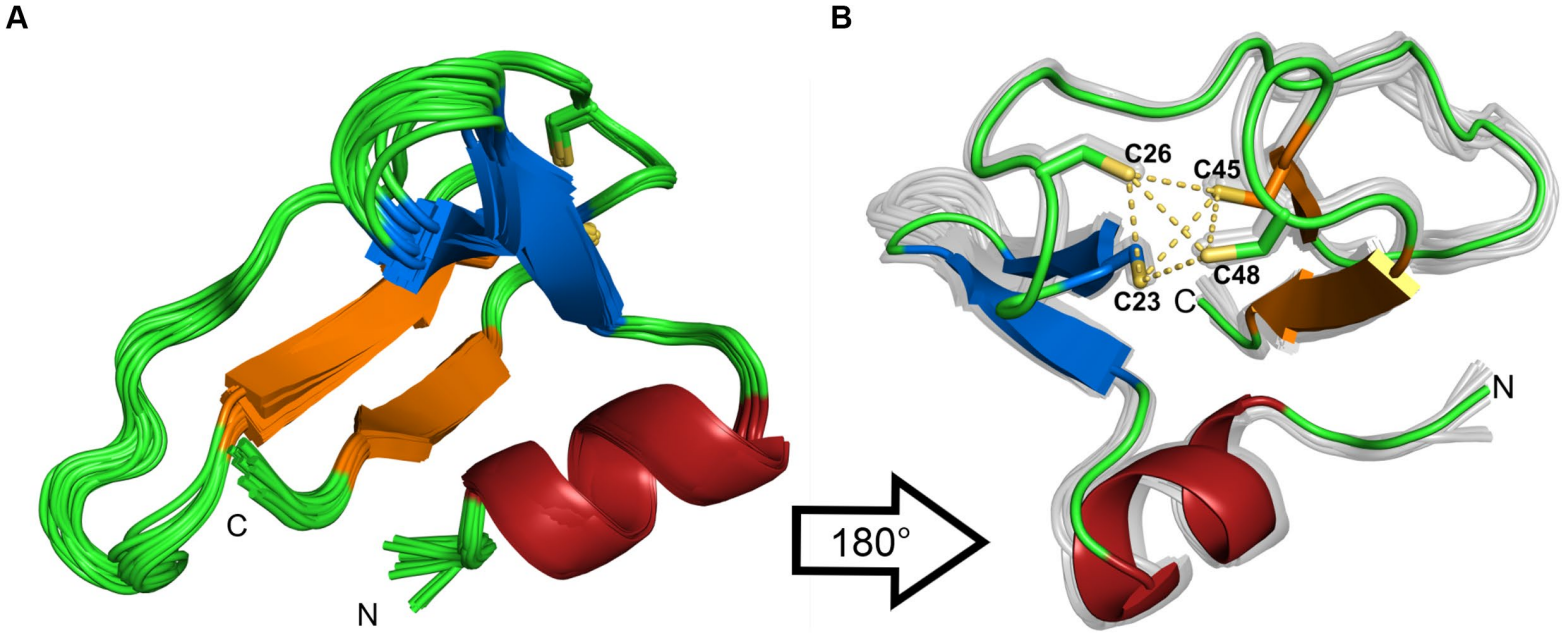
**(A)** Ribbon representation of the 20 lowest-energy structures from the structure calculation. **(B)** 180° rotation of the structure. Additionally, the coordinating sulfur atoms of the zinc-binding pocket are highlighted in yellow. The β-sheets are colored in blue and orange, the α-helix is colored in red. The figures were generated by PyMOL.

The 20 lowest energy calculated structures are shown as a bundle in Figure 4A. The protein has an N-terminal α-helix with several positive charges, followed by a twisted antiparallel β-sheet. The middle part of the protein is comprised of a more flexible part that could be a potential third β-strand to the second anti-parallel β-sheet, which is located at the C-terminus. This is assumed due to some observed cross peaks in the NOESY spectra between K31 and I44, as well as K33 and V42. In addition, TALOS-N predicts a β-strand from K31 to N34. Two cysteines are found after the first β-sheet, which together with two more cysteines from the β-turn of the second β-sheet form a well-defined zinc binding pocket, that can be classified as a zinc-ribbon structure (Krishna et al., 2003).

To rule out dimerization, we compared the experimental rotational correlation time τ_c_ obtained via TENSOR2 (Dosset et al., 2000) with the calculated one from Hydronmr (García et al., 2000). Hydronmr calculates τ_c_ based on a provided structure, while TENSOR2 also takes measured relaxation parameters (R_1_, R_2_, hetNOE) into account. The obtained τ_c_ values are 4.301 ns for Hydronmr and 4.074 ns for TENSOR2. Since the difference between them is small, we can conclude that the protein exists as a monomer.

### Phenotypes of an in-frame deletion mutant

In a previous study 16 deletion mutants of C(P)XCG protein encoding genes were generated, and their phenotypes were characterized (Nagel et al., 2019). The *HVO_0758* deletion mutant exhibited distinctive phenotypes, including a delayed onset of growth in glycerol medium, an inability to swarm, and enhanced biofilm formation in comparison to the wild-type. Prior to further characterizing HVO_0758, we sought to confirm these phenotypes. First, a multiple cycle PCR analysis was used to prove that the deletion was still homozygous (Supplementary Figure S7A). Because *H. volcanii* is highly polyploid, a deletion mutant might still retain a wild-type copy of the genome, potentially reverting to the wild-type over extended storage or cultivation. Furthermore, Northern blot analysis demonstrated the absence of HVO_0758 transcript (Supplementary Figure S7B).

Normally the swarm plate assay is performed in six well plates for 2 days (Nagel et al., 2019). To analyze the behavior of the HVO_0758 deletion mutant upon prolonged incubation, 8 cm Petri dishes were used and the incubation time was considerably extended from 42 h to 192 h (Supplementary Figure 8A). The deletion mutant did not swarm at all for the first 3 days, confirming the lack of swarming during 42 h observed before. However, then the mutant started to swarm with the same velocity as the wild-type. It can be hypothesized that a regulatory step missing in the mutant can be circumvented after prolonged incubation and the chemotaxis pathway can be induced in spite of the missing step.

The enhanced biofilm formation of the *HVO_0758* deletion mutant was verified (Supplementary Figure 8B). After 48 h, the mutant exhibited over twofold greater biofilm formation compared to the wild-type.

Initially, replicating the glycerol medium phenotype proved challenging. Eventually, it was uncovered that the phenotype was contingent on the pre-culture’s growth medium. If the pre-culture was cultivated in glycerol medium, the mutant culture initiated growth upon inoculation without any lag phase, paralleling the wild-type (Supplementary Figure 8C, left panel). In contrast, if the pre-culture was cultivated in a complex medium, the mutant’s lag phase extended by a day relative to the wild-type (Supplementary Figure 8C, right panel).

Next, it was attempted to complement the phenotypes. The phenotype in glycerol medium could be fully complemented (Supplementary Figure S9). In contrast, the biofilm phenotype and the swarming phenotype could not be complemented. We have experienced the partial complementation of multiple phenotypes also with various additional deletion mutants of genes for zinc finger μ-proteins. Possible explanations are discussed below (see Discussion).

### Transcriptome comparison between wild-type and mutant Δ*HVO_0758*

To gain further insight into the potential functions of HVO_0758, an alternative approach involved comparing the transcriptome of the deletion mutant to that of the wild-type. To this end, two cultures of each strain were grown to mid-exponential growth phase (4–5 × 10^8^ cells per ml) in complex medium. Mutant and wild-type grew identically in glucose medium, therefore, indirect effects based on growth rate-differences were prevented. Subsequently, total RNA was extracted, rRNA was depleted and RNA-Seq was used to determine the sequences of the remaining RNAs. The results have been deposited at the Gene Expression Omnibus (GEO)^1^ and obtained the accession No. GSE228855. First, it was analyzed that the *HVO_0758* transcript was indeed missing in the deletion mutant (Supplementary Figure S10A), which was in accordance with the Northern blot analysis (Supplementary Figure S7B).

All genes displaying more than twofold average transcript level difference between the two strains are listed in Supplementary Table S1 together with, e.g., quantitative transcript level difference, protein name, functional classification, and normalized average counts. A total of 78 genes exhibited more than twofold downregulation in the ΔHVO_0758 mutant, while 29 genes experienced more than twofold upregulation. The high number of 108 differentially regulated genes strongly suggests that HVO_0758 has important biological roles in *H. volcanii*.

In many cases not solitary genes were differentially regulated, but rather clusters of contiguous genes displaying co-regulation. Specifically, 15 clusters of co-regulated genes are represented among the 111 differentially regulated genes. The most prominent cluster was cluster 2 with genes of the large motility/chemotaxis gene cluster (mot/che). Notably, 11 out of the 25 genes (HVO_1201- HVO_1225) were downregulated twofold to twentyfold in the deletion mutant, in agreement with the chemotaxis defect described above. Additionally, downregulation was observed for genes responsible for transducer proteins such as MpcT (HVO_0420), and two paralogs of Htr15 (HVO_0555, HVO_3005), even though they reside outside of the mot/che gene cluster but still contribute to chemotaxis. Notably, 29 out of the 78 down-regulated genes encode “conserved hypothetical proteins.” A severe downregulation exceeding fivefold was observed for 11 of these genes, with the most intensely downregulated gene coding for a hypothetical protein. These results indicate that conserved hypothetical proteins might play as yet unknown functions in chemotaxis, or that HVO_0758 regulates additional important processes that have not been detected in the phenotypic analyses of the deletion mutant. Notably, the transcript level of gene *HVO_B0382* encoding a TATA box binding protein (TBP) was fivefold down-regulated. *H. volcanii* contains four TBP paralogs, three are encoded on the major chromosome, and HVO_B0382 is encoded on the minor chromosome pHV3. An analysis of the number of reads using the Integrated Genome Browser visualized the severe downregulation of *HVO_B0382* (Supplementary Figure S10B), which was also verified by Northern Blot analysis (Supplementary Figure S7B). It is tempting to speculate that this TBP paralog might be involved in transcription initiation at several or many of the genes that are downregulated in the HVO_0758 deletion mutants.

Two genes encoding pilins (HVO_2450, HVO_2451) displayed more than twofold upregulation, in agreement with the increased biofilm formation of ΔHVO_0758. The largest up-regulated gene cluster, cluster 16, encompassed 12 genes (green in Supplementary Table S1). This set included genes encoding subunits of nitrite reductase, nitric oxide reductase, a copper-containing oxidoreductase, and halocyanine (HVO_2141, HVO_2147, HVO_2153, HVO_2150). Upregulation of genes for proteins linked to (anaerobic) redox processes could be interpreted as an adaptive response in preparation for biofilm formation, given the diminished oxygen availability within biofilms. Within the 29 up-regulated genes, seven encode “conserved hypothetical proteins.” The remaining up-regulated genes have various annotated functions, yet these functions did not provide insight into any additional biological role of HVO_0758 that could be tested.

Taken together, the phenotypic analysis together with the transcriptome analysis nicely complemented one another, showing that the downregulation of genes encoding the archaella (archaeal flagella) and chemotaxis proteins in the deletion mutant lead to the observed defect in swarming.

### Transcriptome comparison between wild-type and mutant Δ*HVO_2753*

Recently we have reported the detailed analysis of another C(P)XCG zinc finger μ-protein of *H. volcanii*, HVO_2753 (Zahn et al., 2021). Similar analyses were performed as in the present study, including the generation of a deletion mutant, its phenotypic characterization, the heterologous and homologous production of the protein, its biochemical characterization, and the determination of the NMR solution structure. Notably, the deletion mutant also exhibited a swarming defect, similar to the deletion mutant discussed in this study. However, a transcriptome analysis had not been conducted in the former study. The interesting results of the transcriptome analysis of deletion mutant ΔHVO_0758 described above prompted us to address this gap, and a transcriptome analysis of ΔHVO_2753 was performed. Again, RNA was isolated from mid-exponential cultures (two biological replicates). Subsequently, RNA-Seq analysis was carried out as detailed above and in the Methods section. All RNA-Seq results have been deposited at the Gene Expression Omnibus (GEO, see Footnote 1) and obtained the accession No. GSE228855. Supplementary Table S2 contains all genes displaying transcript levels that were more than twofold up- or downregulated in the deletion mutant and the same features as in Supplementary Table S1. In total, 51 genes were down-regulated and 60 genes showed up-regulation. Once more, numerous differentially regulated genes clustered together in contiguous regions. In sum, 15 clusters of differentially co-regulated genes were identified.

Remarkably, all 17 genes that were most down-regulated in the ΔHVO_2753 mutant were localized in the mot/che gene cluster. For these genes the transcript levels were undetectable or extremely low. In fact, the transcript levels of all 25 genes of the HVO_1201-HVO_1225 gene cluster were down-regulated at least threefold. Once more, the transcript levels of additional chemotaxis genes that are situated outside of this gene cluster were also down-regulated, e.g., genes for the transducers Htr15 (HVO_0555), HemAT (HVO_1126), Htr15 (HVO_3005), Htr7 (HVO_1999), and BasB (HVO_0553). Again, the high number of down-regulated motility and chemotaxis genes is in excellent agreement with the observed swarming defect (Zahn et al., 2021). Further examination of the data revealed that among the remaining genes, 14 encode “conserved hypothetical proteins,” once more underscoring the lack of knowledge about the biological functions of many proteins.

Among the up-regulated genes, two gene clusters stand out prominently. The first, cluster 9, contains six genes from the *agl* (archaeal glycosylation) gene cluster, two of which are up-regulated more than fivefold. These *agl* genes encode enzymes that are essential for biosynthesis of two distinct N-linked glycans, which are post-translationally coupled to the surface layer glycoprotein and additional proteins of *H. volcanii* (Pohlschroder and Esquivel, 2015; Eichler, 2020).

The second and notably larger cluster, Cluster 13, includes 20 up-regulated genes. It contains four genes encoding an ABC transporter with the annotated substrate “sugar” (HVO_2031 – HVO_2034), the gene for TrmB (HVO_2035), which has been shown to be a sugar sensor and a transcriptional regulator of sugar ABC transporters in *Thermococcus litoralis* and *Pyrococcus furiosus* (Lee et al., 2003, 2005), and a gene for a GalE sugar epimerase (HVO_2040). The remaining genes encode for seven conserved hypothetical proteins and proteins that are not indicative of sugar metabolism. Nevertheless, together the genes within the up-regulated clusters 9 and 13 suggest that in the ΔHVO_2753 mutant sugar import, sugar metabolism and glycoprotein production are induced.

Table 2 gives an overview of the total numbers of differentially regulated genes, the numbers of up- and down-regulated genes, and several additional features of the transcriptome analyses of the two deletion mutants. Supplementary Table S3 summarizes the genes of the mot/che gene cluster that are down-regulated in the two mutants and the annotated functions of the genes. Taken together, for both deletion mutants the transcriptome analyses and the phenotypic analyses were in excellent agreement.

**Table 2:** Overview of the numbers of more than twofold differentially regulated genes between the wild-type and the in-frame deletion mutants ΔHVO_0758 and ΔHVO_2753.

Class of genes	ΔHVO_0758	ΔHVO_2753
Total No. of >2x regulated genes	108	111
No. of conserved hypothetical genes	40	29
No. of co-regulated gene clusters	19	15
No. of downregulated genes	78	51
No. in the mot/che gene cluster	11	25
No. of up-regulated genes	29	60

The transcript levels were determined via RNA-Seq in rRNA-depleted RNA samples from two biological replicates of cultures in the mid-exponential growth phase.

### Homologous production and purification of HVO_0758

For homologous overproduction in *H. volcanii*, the *HVO_0758* gene was cloned into the shuttle vector pSD1/R1-6, which contains a strong synthetic promoter (Danner and Soppa, 1996). To generate a tagged fusion protein, the sequence for a C-terminal hexahistidine tag was added with one of the oligonucleotides used to amplify the gene. This strategy resulted in the homologous overproduction of a tagged fusion protein, which could be isolated via nickel chelating affinity chromatography. The elution fractions contained a high amount of HVO_0758, endogenous *H. volcanii* proteins that had a stretch of histidines (PitA and Cdc48; Allers et al., 2010), and some proteins that were co-isolated with HVO_0758 (Supplementary Figure S11A). Preparative size exclusion chromatography (SEC) was used as a second purification step to isolate pure monomeric HVO_0758 (Supplementary Figure S11B). The isolated protein was used for biochemical characterizations described below.

### Biochemical characterization of HVO_0758

First, the zinc content of HVO_0758 was quantified. The isolated protein was dialyzed against a low salt buffer, because haloarchaeal proteins typically denature at low salt (Oren, 2008; Gunde-Cimerman et al., 2018). Nevertheless, only a small fraction of 0.2 zinc ions per molecule HVO_0758 could be determined. Therefore, the protein was hydrolyzed by proteinase K, and the zinc quantification was repeated. Notably, 1.0 zinc ions per HVO_0758 molecule were measured, revealing that (1) HVO_0758 is a *bona fide* zinc finger protein, and (2) that zinc binding is largely, but not fully preserved at low salt. This result is in agreement with the NMR results described above, which revealed that at 1 M NaCl the protein had a native structure, while at 0.3 M NaCl the native and a non-native structure co-existed.

**Figure 5:**
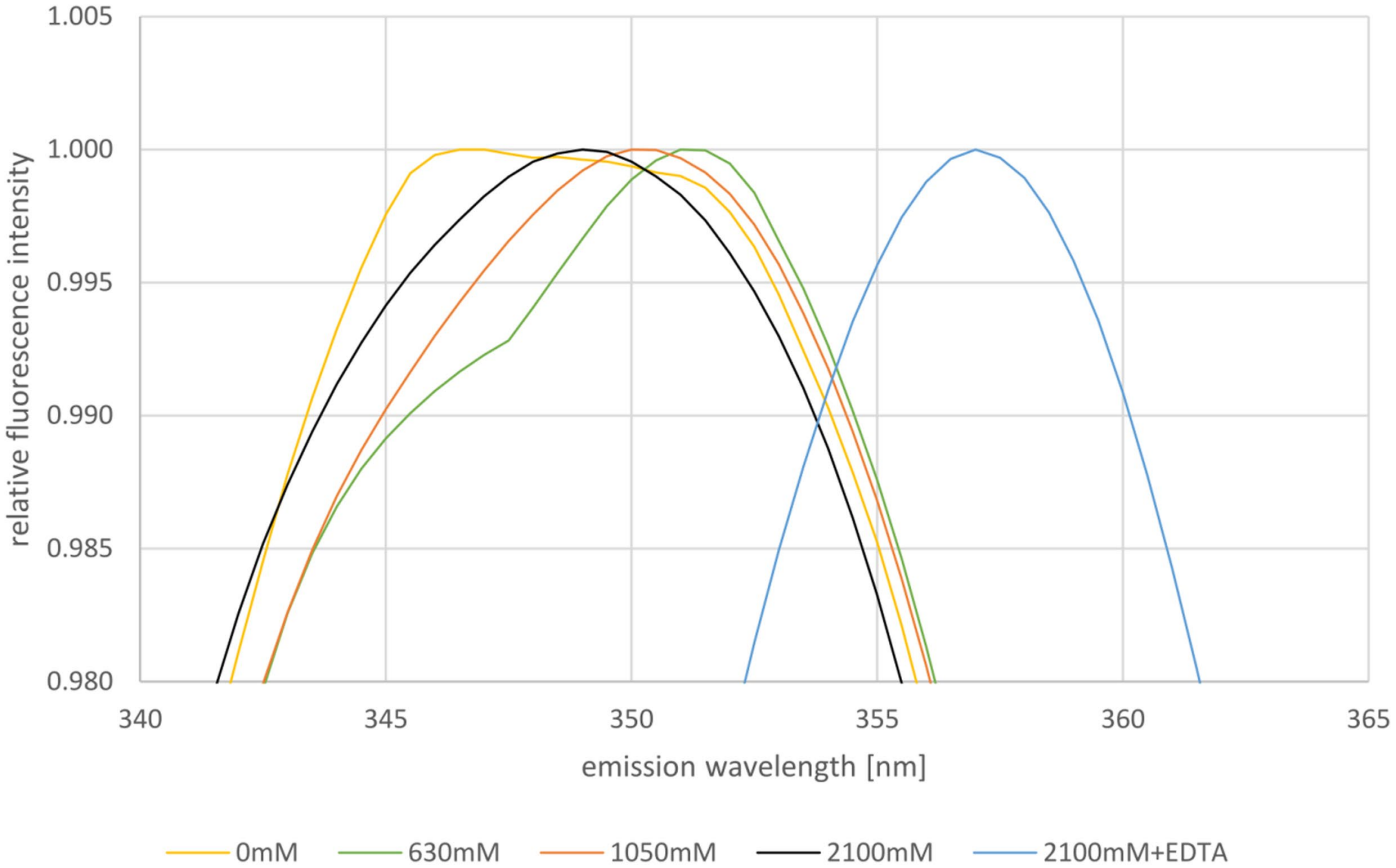
Normalized fluorescence emission spectra of HVO_0758 in buffers containing different NaCl concentrations, as indicated below. The relevant wavelength range from 340 nm to 365 nm is shown. Ten consecutive measurements per sample were performed and averaged. The spectra were normalized to 1 at the respective maximum fluorescence intensity.

Next, the stability of HVO_0758 was studied using tryptophan fluorescence. The maximum of the emission spectrum of tryptophan is around 330 nm to 350 nm and it depends on the hydrophobicity/hydrophilicity of its environment. In a hydrophobic environment the maximum is at a shorter wavelength, in a hydrophilic environment it is at a longer wavelength. Therefore, tryptophan fluorescence is well suited to study the unfolding of proteins (Varejão and Reverter, 2023). HVO_0758 contains one tryptophan near the C-terminus (W52, of 56 aa), which is in a rather hydrophobic environment and can be expected to change the fluorescence properties upon protein denaturation (compare Figure 4). The fluorescence emission spectrum was measured at the optimal NaCl concentration of 2,100 mM as well as reduced concentrations of 1,050 mM, 630 mM, and 0 mM (Figure 5; Supplementary Figure S12). The NMR analysis had revealed that HVO_0758 was stable and had a native fold at 1 M NaCl (see above). Nevertheless, the reduction of the salt concentration from 2,100 mM to 1,050 mM led to a slight red shift of the maximum from 349 nm to 350 nm, underscoring that the tryptophan fluorescence is very sensitive to slight changes in the surrounding of tryptophans (Figure 5). At a NaCl concentration of 630 mM, the maximum was further shifted to 351 nm, and a second maximum around 346 nm appeared. This second maximum became more pronounced in the absence of salt. These results show that at low salt concentrations HVO_0758 has two different conformations, in excellent agreement with the NMR results. Unexpectedly, the maximum of the new conformation was blue-shifted, indicating that W52 moved to a more hydrophobic environment and the protein did not fully unfold to a random coil. The addition of EDTA to the protein at the optimal salt concentration led to a large red-shift to 356 nm, showing that EDTA was able to remove the zinc ion from the protein at room temperature and high salt, and that the protein unfolded in the absence of the zinc ion, again, in agreement with the NMR results.

In another approach the isolated protein was analyzed by mass spectrometry to determine whether or not it carries post-translational modifications. However, the major peak was at 7.301 kDa, exactly the mass of the unmodified protein (Supplementary Figure S13). Two minor peaks with higher masses were observed, which represent the protein with one and with two pairs of oxidized cysteines, respectively. Therefore, the MS analysis did not give any indication of a posttranslational modification, in spite of the high number of hydrophilic and charged amino acids that could potentially be modified.

### Generation and characterization of point mutants

The next approach was the generation of point mutants, with the aim to unravel how the replacement of single amino acids affects zinc binding, folding, stability, and *in vivo* function. First, the four cysteines were replaced by alanines. However, the proteins became very unstable, and it was impossible to isolate even low amounts of protein via nickel chelating affinity chromatography from the mutants, in contrast to the wild-type HVO_0758 (Supplementary Figure S14A).

Next, the following three additional amino acids were selected and single alanine mutants were generated: arginine 9, glutamate 28, and lysine 31. Unfortunately, only very low protein amounts could be isolated from all three point mutants (Supplementary Figure S14B), indicating that all three mutations compromise the half-life of HVO_0758. The protein amounts were too low for the intended biochemical characterizations, and it also inhibited complementation studies. The large effect of all seven point mutations on the intracellular protein concentration led to the decision to terminate mutant studies.

## Discussion

The project of characterizing C(P)XCG proteins from *H. volcanii* is part of the priority program “Small proteins of prokaryotes: an unexplored world” (SPP2002).^2^ Notably, a characterization of the small proteome of *H. volcanii* combined ribosomal profiling with “small protein-optimized mass spectrometry” and led to the identification of 55 novel μ-proteins (Hadjeras et al., 2023). Only seven of these were found with both methods, while 40 were found solely by ribosomal profiling and 8 solely by peptidomics. The set of novel proteins overlapped, but was not identical to the set of novel proteins found in a second ribosomal profiling study with *H. volcanii* (Gelsinger et al., 2020). Together, these studies underscore that no experimental approach alone can unravel the full diversity of the μ-proteome of a species of interest.

The current *H. volcanii* project was chosen because 8% of all archaeal μ-proteins contain two C(P)XCG motifs and are putative zinc finger proteins, while that is only true for 1.5% of bacterial μ-proteins (Tarasov et al., 2008). A first attempt to clarify whether or not C(P)XCG proteins are important for archaeal biology and gain an insight into putative functions, 16 *in-frame* deletion mutants have been generated and phenotypically characterized, including *HVO_0758* (Nagel et al., 2019). Currently, more than 30 C(P)XCG genes of *H. volcanii* have been deleted, and the majority exhibited phenotypic differences from the wild-type. Five genes could not be deleted and are thus essential, underscoring that C(P)XCG proteins fulfill important functions in *H. volcanii* and probably other archaea (data not shown). The aim is to select very few of these 43 C(P)XCG μ-proteins for a detailed analysis.

The first selected protein was HVO_2753, because it is the only *H. volcanii* protein with four C(P)XCG motifs, and its characterization has recently been published (Zahn et al., 2021). The next selected protein was HVO_0758, which was chosen based on the interesting phenotype of the *in-frame* deletion mutant, i.e., a defect in swarming, enhanced biofilm formation, and a lag phase after inoculation in synthetic medium with glycerol (Nagel et al., 2019). The two proteins turned out to be very different, and, therefore, at the end of this discussion an overview of similarities and differences will be given.

HVO_0758 could be produced heterologously in *E. coli*, could be purified, and turned out to be in a folded conformation. This is not typical for μ-proteins from various prokaryotic species. In an overview study in the framework of SPP2002 it was attempted to produce and characterize 27 μ-proteins from nine bacterial and archaeal species (Kubatova et al., 2020). It turned out that only four of these proteins were folded and one protein was partially folded, whereas the majority of proteins were in the molten globule state, were totally unfolded, or could not be produced. Various reviews summarize the specific challenges in the heterologous or homologous production of μ-proteins, in contrast to average-sized proteins (Storz et al., 2014; Orr et al., 2020; Cassidy et al., 2021; Steinberg and Koch, 2021; Tharakan and Sawa, 2021; Chen et al., 2022; Gray et al., 2022; Leong et al., 2022; Schlesinger and Elsässer, 2022; Weidenbach et al., 2022; Zhang et al., 2022; Dong et al., 2023; Hassel et al., 2023).

The heterologously produced HVO_0758 turned out to have a fully folded conformation at 1 M NaCl, but to be a mixture of a native and a non-native conformation at 0.3 M NaCl. A mixture of two conformations was also observed with the homologously produced protein at low salt using tryptophan fluorescence. Together, these results indicate that HVO_0758 is not stable at low salt concentrations. However, the tryptophan fluorescence data as well as the zinc assay showed that it is not fully denatured at low salt. This is in contrast to other haloarchaeal proteins, which denature at low salt (Oren, 2008). Therefore, it seems that the complexation of four cysteines by the zinc ion protects HVO_0758 from denaturation, which typical haloarchaeal proteins experience at low salt. This might turn out to be a general advantage of haloarchaeal zinc finger proteins.

The zinc content of HVO_0758 was determined with two independent methods. On the one hand zinc was titrated to zinc-free HVO_0758, and the NMR analyses revealed that the addition of one zinc equivalent per HVO_0758 protein resulted in the generation of the native conformation. On the other hand, quantification of zinc ions after the proteolysis of isolated HVO_0758 revealed that one zinc ion had been bound to the protein prior to proteolysis. Together, these results showed that HVO_0758 is a *bona fide* zinc finger protein. This needs to be verified experimentally, because the C(P)XCG motifs are indicative for zinc binding, but do not prove it. For example, HVO_2753 has four C(P)XCG motifs, but binds only one zinc ion, while the second predicted “zinc finger” is zinc free (Table 3). Up to now 10 haloarchaeal μ-proteins with C(P)XCG motifs have been characterized, and four of them did not bind zinc, in contrast to the bioinformatics prediction (data not shown). The loss of metal binding in metal binding proteins by mutation, yielding folded and functional metal-free descendants, is not uncommon in the evolution of metal binding proteins (Torrance et al., 2008). It seems that in these cases the protein structure is so stabilized by alternative interactions, that mutations can lead to the loss of zinc binding without the concomitant loss of the protein structure or function.

**Table 3:** Predicted and experimentally quantified zinc content of the proteins HVO_0758 and HVO_2753.

Predicted	Experimentally quantified before proteolysis after proteolysis		
HVO_0758	1	0.17	1.0
HVO_2753	2	0.20	0.7

The NMR solution structure of HVO_0758 has been solved (Figure 4). In the Protein Database (PDB) no protein with a similar structure could be found, therefore, the structure will be helpful as template for future structure determinations of structure predictions (e.g., with AlphaFold) of homologous proteins and non-homologous proteins of similar structure.

HVO_0758 is characterized by an N-terminal alpha-helix of about 10 aa, which sticks out and looks like an interaction domain. It contains several positive amino acids, therefore, it is tempting to speculate that HCO_0758 might bind DNA or RNA. The unusual pI of 7.6 (for haloarchaeal proteins) and the differential regulation of many genes might point in the same direction. Unfortunately, attempts to co-isolate DNAs or RNAs or to measure DNA binding were unsuccessful until now.

The protein part C-terminal of the alpha-helix is folded around the zinc finger (Figure 4). The zinc binding seems to be of utmost importance for the protein structure, because in all four C- > A mutants no protein could be detected (Supplementary Figure S14A), and because the native structure was lost upon addition of EDTA (NMR, tryptophan fluorescence). This is not always the case for zinc fingers in other proteins. For example, bacterial Ros proteins, which are transcriptional regulators, typically contain a C_2_H_2_ zinc finger (Malgieri et al., 2015). However, in a minority of homologs one of the two cysteines is replaced by another amino acid, nevertheless, these proteins with an incomplete zinc finger motif are able to bind zinc and DNA, and lose both features upon addition of EDTA (Baglivo et al., 2009). In addition, a C82D mutant of the well-studied Ros protein from *Agrobacterium tumefaciens* was generated, and also the mutant protein kept the ability to bind zinc and DNA (Baglivo et al., 2009). However, typically four cysteine/histidine ligands are essential for zinc binding, like in HVO_0758.

The phenotypic analyses revealed that the Δ*HVO_0758* mutant exhibited an extended lag phase after cells pre-grown in complex medium were inoculated in synthetic medium with glucose. This seems to indicate that HVO_0758 might be important for the transition from one carbon source to another. Transitions between different conditions occur with extremely different velocities in *H. volcanii*. Whereas upregulation of the gene for the tryptophanase (HVO_0009) can be observed already 2 min after tryptophane addition to the medium (unpublished results), it takes *H. volcanii* about 20 h to adapt to a low salt medium containing 0.7 M NaCl (Jantzer et al., 2011). In an earlier study we have shown that *H. volcanii* exhibits a lag phase of about 1 h when cells pre-grown on casamino acids were switched to glucose as sole carbon and energy source (Zaigler et al., 2003). During this lag period more than 30 genes were transiently induced, which had identical transcript levels during steady state growth in both media. Some or all of the encoded proteins can be hypothesized to be important for the transition process, but not for steady states.

Deletion of both genes, *HVO_0758* and *HVO_2753*, led to a complete loss of swarming during the first days, indicating that both zinc finger μ-proteins are essential for motility and chemotaxis. In agreement with the phenotype the analyses of the transcriptomes of both mutants revealed that various genes of the large mot/che gene cluster are considerably down-regulated (Supplementary Table S3), nicely explaining the phenotype.

The Δ*HVO_0758* mutant showed an enhanced biofilm formation, while the Δ*HVO_2753* mutant had a defect in biofilm formation. In agreement with this difference, there was no overlap of up-regulated genes in the transcriptome analyses. In the transcriptome of Δ*HVO_0758* genes for redox enzymes were induced, e.g., for nitrite reductase and nitrite oxide reductase. This can be speculated to be a pre-adaptation for biofilm formation, because oxygen availability is limited in biofilms. Also, two genes for pili were induced, which is in agreement with the essential roles of pili in biofilm formation of *H. volcanii* (Pohlschroder and Esquivel, 2015; Esquivel et al., 2016). In contrast, genes for sugar metabolism and glycan biosynthesis were up-regulated in the Δ*HVO_2753* mutant. The *agl* (archaeal glycosylation) genes form a very large cluster in the genome of *H. volcanii* (HVO_2046 – HVO_2061). In *H. volcanii* the N-glycosylation pathways have been well-studied, and targets for N-glycosylation are, e.g., the major surface layer protein and pili (Esquivel et al., 2016; Kandiba et al., 2016; Tamir and Eichler, 2017; Pohlschroder et al., 2018; Shalev et al., 2018; Eichler, 2020; Schulze et al., 2021). Future studies are needed to unravel how an enhanced N-glycosylation of the S-layer and/or the pili might interfere with swarming and/or biofilm formation.

For the *HVO_0758* deletion mutant, the growth deficit in glycerol medium could be complemented (Supplementary Figure S9), while the swarming phenotype and the biofilm phenotype could not be complemented (data not shown). Partial complementation was also observed for various additional mutants. The following reasons can possibly explain the failure to complement some of the phenotypes: (1) the His_6_-tag can prevent interactions with other molecules that are essential for function, (2) the His_6_-tag (more than 10% of the size μ-proteins) might influence the overall folding of a protein and thereby change the binding affinity to other molecules, (3) the His_6_-tag might influence the half life of the protein, and (4) the function maybe sensitive to the intracellular concentration, which differs from the native concentration when the gene is expressed from a plasmid instead of the native chromosomal site.

## Conclusion and outlook

The present study represents an in-depth analysis of the μ-protein HVO_0758, which contains two C(P)XCG motifs and thus had the potential to be a zinc finger protein. The protein was produced heterologously in *E. coli* and homologously in *H. volcanii* and the isolated protein was characterized with various approaches, including a quantitative zinc assay, tryptophan fluorescence, and different NMR measurements. An *in-frame* deletion mutant and seven point mutants were generated. HVO_0758 was shown to be a *bona fide* zinc finger protein and binds one zinc ion per protein molecule. The NMR solution structure revealed that it is comprised of an N-terminal alpha helix with several positively charged amino acids that is placed on top of a globular core, which is stabilized by the zinc finger. HVO_0758 is the second *H. volcanii* C(P)XCG μ-protein that has been thoroughly characterized. Table 4 summarizes the similarities and differences of the two proteins, HVO_0758 and HVO_2753.

**Table 4:** Comparison of characteristic features of HVO_0758 and HVO_2753.

Feature	HVO_0758	HVO_2753
Conserved in	Haloferax	Haloarchaea, methanogenic Archaea
No. aminoacids	56	59
pI value	7.6	6,7
Stability at low salt	partly	fully
No. C(P)XCG motifs	2	4
Predicted zinc fingers	1	2
Zinc ions bound per protein molecule	1	1
Zinc removal upon EDTA addition	yes	no
Cysteines essential for folding	yes	yes
Unordered N-Terminus	no	yes
Helix on top of globular core	yes	no
Phenotype of a deletion mutant:		
Growth in many media	normal	normal
Growth on glycerol	lag phase	normal
Swarming	loss	loss
Biofilm formation	increase	decrease
RNA-Seq: down-regulated	mot/che genes	mot/che genes
RNA-Seq: up-regulated	redox proteins	sugar metabolism
		Glycan synthesis

Of course it would be of interest to identify binding partners of HVO_0758, which might shed light on the molecular mechanism of regulating swarming and biofilm formation. However, initial attempts to co-isolate proteins, DNA, or RNA molecules that bind HVO_0758 specifically did not lead conclusive results, and thus they are omitted in this presentation. The experimental design of the co-isolation approaches will be optimized and the results will be published in the future.

## Materials and methods

### Databases and bioinformatics analyses

All gene and protein sequences derived from the *H. volcanii* genome were obtained from the HaloLex database (Pfeiffer et al., 2008).^3^ To visualize the transcript levels of *HVO_0758* and further selected transcripts, the respective results of a RNA-Seq and a dRNA-Seq study (Babski et al., 2016; Laass et al., 2019) were visualized using the Integrated Genome Browser (Freese et al., 2016).

Homologous sequences of the *HVO_0758* gene were searched for using NCBI BlastP (Altschul et al., 1990). The top 100 hits were retrieved and a multiple sequence alignment was generated using the ClustalOmega program at the EMBL-EBI site (Sievers et al., 2011).

### Strains, media, and culture conditions

All strains generated in this study are derived from the *H. volcanii* H26 strain which also served as the wild-type control. The strains were grown in complex medium or synthetic media with different carbon and energy sources as described previously (Allers et al., 2004; Dambeck and Soppa, 2008; Jantzer et al., 2011). All strains with expression plasmids based on pSD1 were grown in the presence of Novobiocin (0.5 μg/mL; Danner and Soppa, 1996).

The *E. coli* strain XL1-Blue MRF’ (Agilent Technologies, Waldbronn, Germany) was used for cloning and construction of all plasmids used in this study (Green and Sambrook, 2012).

### Heterologous production in *Escherichia coli*, purification, and characterization

The ORF for HVO_0758 was introduced into the pE-SUMO vector via BsaI and XbaI restriction sites. This puts the protein directly behind the C-terminal double glycine of the SUMO protein, leading to a fusion protein of SUMO protein and the protein of interest with a His_6_-tag on the N-terminus of the SUMO protein.

The plasmid carrying the ORF was used to transform BL21(DE3) *E. coli* cells, which were subsequently cultivated in M9 minimal medium enriched with ^15^N-NH_4_Cl alone or together with ^13^C-Glucose for isotope-labeled samples. Ampicillin was added to the M9 medium (final concentration 1 mM) to ensure presence of the plasmid in the *E. coli* cells. The cultures were incubated at 37°C until turbidity reached an OD_600_ of 0.6. Protein overproduction was then induced by addition of 1 mM IPTG. The cultures were further incubated overnight and then harvested by centrifugation at 5000 rpm and 4°C for 15 min. Cell pellets were either flash-frozen and stored at −80°C or directly resuspended in buffer (25 mM BisTris pH 7, 300 mM NaCl, 5 mM 2-mercaptoethanol, 100 μM ZnCl_2_) together with protease inhibitor (cOmplete^™^, Roche, Germany; one tablet per liter) for purification. The cells were lysed by french press and then centrifuged (16,000 x g, 45 min, 4°C) to remove cell debris. The supernatant was loaded onto a HisTrap for affinity purification and subsequently eluted at ~20% of an imidazole gradient up to 500 mM imidazole. Cleavage of the SUMO protein to obtain the pure desired protein was achieved by addition of Ulp1 (SUMO protease) during dialysis against buffer without imidazole. The protein was then applied to a HisTrap again and pooled in the flow-through, while the cleaved His-tagged SUMO protein bound to the column. In the final purification step, size-exclusion chromatography was carried out to obtain >95% pure protein. After every step, SDS-PAGE was carried out for relevant fractions of the columns to determine identity and purity of the protein.

### NMR spectroscopic experimental data

NMR measurements were carried out in buffer containing 25 mM BisTris pH 7, 1 M NaCl, 5 mM 2-mercaptoethanol, 100 μM ZnCl_2_ and 5% D_2_O at 298 K. In order to reference spectra, 1 mM DSS was added and the proton methyl signal of the trimethylsilyl group set to 0.00 ppm. ^13^C and ^15^N referencing was done according to Wishart (2011). All used NMR spectrometers were manufactured by Bruker and equipped with cryogenic probeheads with z-axis gradient ^1^H{^13^C,^15^N}. Spectrometer frequencies ranged from 600 to 900 MHz.

Backbone assignment was done in a 2D ^1^H^15^N HSQC with 3D experiments HNCO, HN(CA)CO, HNCACB and HN(CO)CACB. Side-chain assignment was done in a 2D ^1^H^13^C HSQC with 3D experiments H(CCO)NH, CC(CO)NH, HCCH-TOCSY, HCCH-COSY and ^15^N-TOCSY-HSQC. The aromatic assignment was done in a 2D ^1^H^13^C TROSY together with a 3D ^1^H^1^H^13^C NOESY-SOFAST-HMQC (mixing time 100 ms).

For the NOE-based distance restraints of the structure calculation 3D ^1^H-^1^H-^15^N-NOESY-HSQC and ^1^H-^1^H-^13^C-NOESY-HSQC spectra (mixing time for both 120 ms) were measured. A 3D HNHA spectrum was measured for the calculation of ^3^J_HNHα_ coupling constants (Vuister and Bax, 1993).

A temperature series was measured with a series of 1D ^1^H and 2D ^1^H^15^N HSQC spectra ranging from 278 K to 333 K in 5 K increments. Temperature coefficients were determined from this by plotting the amide proton chemical shift perturbations against the temperature with a linear fit (Baxter and Williamson, 1997).

2D ^15^N-ZZ-exchange experiments were carried out to confirm that two conformations of the protein were present at low NaCl concentrations. Mixing times were varied from 100 ms to 800 ms. Cross peaks could be observed best at 200 ms.

Relaxation experiments were performed with a ^15^N-labeled sample on a 600 MHz spectrometer. The ^15^N T_1_ relaxation time was obtained from a pseudo-3D spectrum measuring a series of ^1^H^15^N spectra with increasing relaxation delays (20, 60, 100, 200, 400, 600, 800, 1,200, 1,500 and 1800 ms). The ^15^N T_2_ relaxation time was obtained from a pseudo-3D spectrum as well (delays: 16.96, 33.92, 67.84, 135.68, 169.60, 203.52, 237.44 and 271.36 ms). {^1^H}-^15^N-hetNOEs were measured as the ratio of signal intensities from two spectra recorded with and without amide proton saturation. Relaxation parameters were used to determine the S^2^ order parameter and the experimental rotational correlation time both with TENSOR2.

All spectra were recorded and processed using TopSpin (different versions). Assignment was carried out using NMRFAM-SPARKY 1.470 (Lee et al., 2015).

### Structure calculation

The structure calculation was performed with CYANA 3.98.13. Three 3D NOESY spectra were used as input for the fully automated NOE cross-peak assignment: ^1^H^1^H^15^N-NOESY-HSQC, ^1^H^1^H^13^C-NOESY-HSQC spectra (mixing time for both 120 ms) and ^1^H^1^H^13^C-NOESY-SOFAST-HMQC (mixing time 100 ms). Peaks in the NOESY spectra were selected using the restricted peak picking of NMRFAM-SPARKY and manually checked and corrected. Peak shift tolerances were set to 0.02 ppm for protons and carbon atoms and 0.2 ppm for nitrogen atoms. Additional restraints included hydrogen bond distances derived from NOESY analysis, ^3^J_HNHα_ coupling constants from the 3D HNHA spectrum and dihedral angles from the TALOS-N prediction. Furthermore, the zinc-binding pocket was defined by setting lower and upper distance restraints between the four sulfur atoms of the coordinating cysteine residues to 3.65 Å and 4 Å, respectively. These distances were chosen by referencing the structures of other zinc-binding proteins. The restraints were only set after we saw that the binding pocket was also present in the calculated structure just through NOESY contacts. A standard protocol was used with 200 initial steps, 15,000 refinement steps and 20 final structures per iteration. The final bundle consisting of the lowest energy structures was deposited in the pdb (ID 8Q5B) and bmrb (ID 34844).

### Generation of an *in-frame* deletion mutant

For the construction of all in-frame deletion mutants, the established Pop-In-Pop-Out method was applied (Allers et al., 2004; Hammelmann and Soppa, 2008). The oligonucleotides for the generation of the deletion mutant of HVO_0758 are listed in Supplementary Table S4. As *H. volcanii* is a polyploid organism with about 20–30 copies of the major chromosome (Breuert et al., 2006; Maurer et al., 2018), it happens sometimes that after the Pop_Out selection not all copies of the wild-type chromosome are replaced by the deletion variant of the respective gene, but that one or a few copies remain and the respective clones are heterozygous clones. Therefore, the homozygosity of the deletion mutant was verified by multicycle PCR using isolated genomic DNA as template, and the absence of the *HVO_0758* transcript was verified via Northern blot analysis.

### Phenotypic characterization: growth analysis

Growth analyses were performed in round-bottom microtiter plates as described previously (Jantzer et al., 2011). A pre-culture was grown in complex medium, harvested, washed once in basal salts and resuspended in basal salts to an OD_600_ of 0.75. For each condition, 140 μL medium was mixed with 10 μL cell suspension, resulting in an OD_600_ of 0.05 at the start of the growth experiment. The cultures were grown on a Heidolph Titramax 1,000 rotary shaker (Heidolph, Schwalbach, Germany) with 1,100 rpm at 42° C. The OD_600_ was determined frequently using the microtiter plate photometer Spectramax 340 (Molecular Devices, Ismaning, Germany). Growth curves were obtained from average values of at least three biological replicates and their standard deviations.

### Phenotypic characterization: swarming assay

The swarming assay was in general performed as described previously (Nagel et al., 2019). Twenty milliliter of synthetic glucose medium with 0.3% (w/v) agar was filled into petri dishes (Sarstedt, Nümbrecht, Germany) 1 day prior to start of the assay. The cells from pre-cultures in the respective medium were harvested by centrifugation and resuspended in basal salts to an OD_600_ of 20. Four microliter of the cell suspension was injected deeply into the swarm agar in the center of the plates, because *H. volcanii* is swarming only at low oxygen concentrations. The plates were incubated for several days at 42°C in a Styrofoam box together with a cup of water to inhibit drying. The swarming diameter was measured and pictures were taken every 24 h.

### Phenotypic characterization: biofilm formation

For the biofilm assay, cultures were grown in synthetic medium with glucose to the mid-exponential growth phase. The OD_600_ was measured, cells were pelleted by centrifugation and resuspended in fresh glucose medium to an OD_600_ of 0.5. For biofilm formation, 24 well microtiter plates were used (Sarstedt, Nümbrecht, Germany).

The biofilm assay consists of several steps, i.e., formation of a biofilm, removal of planktonic cells, fixation and staining of adherent cells, destaining and photometric quantification of the supernatant. The assay has been performed as described previously by Legerme et al. (2016), with some modifications. To this end, 2 mL of cell suspension was transferred into each well, and the plates were incubated without shaking at 42°C for 48 h.

After that, the supernatant was removed, and 1,000 μL of fixing solution [2% (w/v) acetic acid] was given into each well, and the plate was incubated for 5 min at room temperature. The supernatant was removed, and the plate was dried for 1–2 h at 37°C until all the liquid has evaporated. After that, 500 μL staining solution [0.1% (w/v) crystal violet] was given into each well, and it was incubated for 10 min at room temperature. After that, the supernatant was removed, and the wells were washed three times with 3 mL distilled water very carefully to not detach formed biofilm. Subsequently, 2 mL of destaining solution [10% (v/v) acetic acid, 30% (v/v) methanol] was given into each well, and the plate was incubated for 10 min at room temperature until all crystal violet bound in the biofilm was solved homogeneously in the supernatant. Then, 100 μL of the supernatant of each well was transferred into a new 96-well microtiter plate, and the OD_600_ was recorded with a microtiter plate photometer (Spectramax 340, Molecular Devices, San Jose, CA, United States). A negative control (medium without cells) was included in the assay, and its value of the destaining solution (about 0.05) was subtracted from the values of all tested strains.

### Generation of the point mutations of HVO_0758

Point mutants of HVO_0758_NHis and HVO_0758_CHis were created via site-directed mutagenesis according to the protocol of the QuikChange II Site-Directed Mutagenesis Kit (Agilent Technologies, Santa Clara, United States). The oligonucleotides used for site-directed mutagenesis are listed in Supplementary Table S4. Since pSD1-R1/6 is too large for this approach (>10 kbp), the native gene along with an either N- or C-terminal His-tag was first subcloned into the vector pSK(+). After site-directed mutagenesis, the mutated gene was excised with KpnI and either NdeI or NcoI and ligated with the vector pSD1-R1/6 linearized with the same enzymes. The sequences of the mutated gene versions were verified by sequencing (GATC/Eurofins; eurofinsgenomics.eu/en/custom-DNA-sequencing).

### Northern blot analysis

The expression levels of selected genes were analyzed by Northern blot analysis.^4^ *H. volcanii* cultures were grown in synthetic glucose medium to mid-exponential growth phase (about 4–6 × 10^8^ cells/ml). Cells were harvested by centrifugation, and total RNA was isolated using phenol/chloroform extraction. Equal amounts of total RNA from each sample (usually 2-5 μg) were separated on a denaturing formaldehyde agarose gel. The RNA was transferred by capillary blotting onto a nylon membrane, and fixed by UV-cross-linking. Digoxigenin-labeled DNA probes were generated by PCR using DIG-dUTP and a dNTP mix with reduced dTTP concentration. The primers for probe generation are listed in Supplementary Table S4. Hybridization was performed overnight at 50°C. The membrane was washed twice with 2 × SSC/0.1% SDS and twice with 1× SSC/0.1% SDS. The probes were detected using an anti-DIG antibody coupled to alkaline phosphatase and the chemiluminescence substrate CDP star according to the instructions of the manufacturer (Roche, Mannheim, Germany). The signals were visualized with Xray films (GE Healthcare), and the sizes were analyzed with the size marker ‘RiboRuler Low/High Range RNA Ladder’ (Thermo Fisher Scientific).

### Transcriptome analysis using RNA-Seq

Cultures were grown to mid-exponential growth phase (4–6 × 10^8^ cells per ml) in glucose medium, cells were harvested, and total RNA was isolated as described above. Ribosomal RNAs were depleted from 1 μg of non-degraded DNase I-digested total RNA by subtractive hybridization using the Pan-Archaea_riboPOOL kit (siTOOLs, Germany) according to the manufacturer’s protocol using Dynabeads MyOne Streptavidin T1 beads (Invitrogen) and then subjected to cDNA libraries preparation.

cDNA libraries were prepared at Vertis Biotechnologies AG (Freising, Germany) using the Adapter ligation method. Briefly, the rRNA-depleted RNA samples were first fragmented using ultrasound (2 pulses of 30 s each at 4°C) and then directly used for cDNA synthesis. First, an oligonucleotide adapter was ligated to the 3′ end of the RNA molecules. First-strand cDNA synthesis was performed using M-MLV reverse transcriptase and the 3′ adapter as a primer. The first-strand cDNA was purified and the 5’ Illumina TruSeq sequencing adapter was ligated to the 3′ end of the antisense cDNA. The resulting cDNA was then PCR-amplified to about 10–20 ng/μl using a high fidelity DNA polymerase (12 PCR cycles). The cDNA was purified using the Agencourt AMPure XP kit (Beckman Coulter Genomics) and was analyzed by capillary electrophoresis. The primers used for PCR amplification were designed for TruSeq sequencing according to the instructions of Illumina. The following adapter sequences flank the cDNA inserts: TruSeq_Sense_primer: (NNNNNNNN = i5 Barcode for multiplexing) 5′-AATGATACGGCGACCACCGAGATCTACAC-NNNNNNNN-ACACTCTTTCCCTACACGACGCTCTTCCGATCT-3′.

TruSeq_Antisense_primer: (NNNNNNNN = i7 Barcode for multiplexing) 5′-CAAGCAGAAGACGGCATACGAGAT-NNNNNNNN-GTGACTGGAGTTCAGACGTGTGCTCTTCCGATCT-3′.

cDNA libraries were pooled on an Illumina NextSeq 500 high-output flow cell and sequenced in single-end mode (75 cycles) with 10 million reads per RNA-seq library (at the Core Unit SysMed at the University of Würzburg). Raw sequencing reads in FASTQ format and normalized coverage files are available via Gene Expression Omnibus (GEO, see Footnote 1) under accession number GSE228855.

### Bioinformatic analysis of the RNA-seq results

To assure high sequence quality, Illumina reads were quality and adapter trimmed via Cutadapt (Martin, 2011) version 1.16.1 using a cutoff Phred score of 20 (command line parameters: -quality-cutoff = 20 -m 20 -a AGATCGGAAGAGCACACGTCTGAACTCCAGTCAC). After trimming, the reads were mapped to the reference genome (*H. volcanii* DS2) using STAR (Dobin et al., 2013; Dobin and Gingeras, 2015) version 2.7.5b. Mapped reads were subsequently counted for all annotated genes using featureCounts (Liao et al., 2014) version 1.6.2. The quality of the raw data and each preprocessing step was assessed using multiQC (Ewels et al., 2016) version 1.6. MultiQC was used to aggregate statistics for featureCounts and fastQC version 0.11.6 into a clear html report.^5^

To facilitate visualization in a genome browser, HRIBO (Gelhausen et al., 2021) version 1.6.0 was used to generate perstrand genomic coverage plots for each library indicating the number of mapped reads per nucleotide. The used annotations for *H. volcanii* DS2 was retrieved from NCBI. The coverage files were visualized using the Integrated Genome Browser IGB (Freese et al., 2016).

### Differential expression analysis

Differential expression analysis for *Haloferax volcanii* DS2 H26 RNA-seq libraries was conducted using DESeq2 (Love et al., 2014) version 1.18.1. Significant results were retrieved by applying a cutoff to the log2 fold change [abs(log2FC) > 2] and the adjusted value of *p* < 0.05 for all output files. Additionally, plots for visualization and quality control were generated. These include heatmaps of the normalized read count correlation, principal component analysis (PCA) and MA-plots for each analyzed contrast.

### Homologous production and native purification

For the homologous overproduction of HVO_0758 the respective gene was cloned into the expression vector pSD1-R1/6 containing a strong constitutive promoter (Danner and Soppa, 1996). The codons for a N-terminal His_6_-tag were added with one of the primers (Supplementary Table S4), so that a fusion protein was produced, which could be isolated with nickel chelating chromatography. The sequence of the resulting plasmid was verified by sequencing, and it was used to transform the *HVO_0758* deletion mutant. The plasmid complemented the phenotype in glycerol medium, and, thus, it was guaranteed that the produced protein was folded and functional *in vivo*. The production strain was grown in complex media with 0.5 μg/mL novobiocin overnight. Fivehundred milliliter of complex medium with 0.5 μg/mL novobiocin was then inoculated with the pre-culture to a start OD_600_ of 0.005. The production culture was grown for 24 h and the cells were harvested by centrifugation (6,500 g, 30 min, 4°C). IMAC was used as a first purification step. The pellet was suspended in 5 mL binding buffer (2.1 M NaCl, 20 mM HEPES pH 7.5, 20 mM imidazole, 1 mM PMSF) and the cells were lysed via sonication on ice. Cell fragments were pelleted by centrifugation (4,000 g, 30 min, 4°C) and the cleared lysate was mixed with Chelating Sepharose^®^ Fast Flow (GE Healthcare) beads which had been charged with Ni^2+^ ions and suspended in binding buffer. The beads were washed four times with binding buffer to remove unspecifically bound proteins. The His-tagged HVO_0758 as well as other histidine-rich proteins were eluted with elution buffer (2,1 M NaCl, 20 mM HEPES pH 7.5, 300 mM imidazole).

Size exclusion chromatography was used as a second purification step in order to separate other specifically-bound proteins from the target protein. Elution fractions of the first purification step were loaded on a SuperDex 75 Increase (10/30) FPLC column (GE Healthcare) with 2.1 M NaCl, 20 mM HEPES pH 7.5 as mobile phase and a flow rate of 0.4 mL/min. A standard curve was generated with a chromatogram of a mixture of the proteins aprotinin (6.5 kDa), RNAse A (13.7 kDa), Ovalbumin (44 kDa) and Conalbumin (57 kDa).

Samples of the different steps of the IMAC as well as the SEC were analyzed via Tricine-SDS-PAGE (Schägger and von Jagow, 1987).

### Fluorimetric zinc quantification

To quantify the bound zinc of the protein, the highly zinc-specific fluorophore ZnAF-2F was used (Hirano et al., 2002). HVO_0758 was purified as described above. Protein concentration was determined via UV-absorption at 280 nm ( $\epsilon_{280}$ =6,990 M^−1^ · cm^−1^) and the experiment was performed as described previously (Zahn et al., 2021). In short, 1 μM Protein was dialyzed against 25 mM NaCl and 20 mM HEPES, pH 7.5, and incubated with proteinase K (100 μg/mL) over night at 37°C to release the bound zinc. 3 μM of the fluorophore was added and the measurements were performed on a microtiter plate fluorimeter (ClarioStar, BMG LabTech, Ortenberg, Germany) with an excitation wavelength of 492 nm and detection at 517 nm. Four technical replicates were used for each biological replicate as well as the standard curve, which was generated with solutions containing 0, 0.5, 1 and 2 μM ZnSO_4_.

### Stability analysis via tryptophane fluorescence

Fluorescence spectroscopy was performed in order to analyze the stability of HVO_0758_CHis in buffers containing various salt concentrations. 1 mg/mL of purified protein was either diluted in or dialyzed against a buffer containing 20 mM HEPES, pH 7.5, lowering the NaCl concentration to 1,050 mM, 630 mM or 0 mM, respectively. One measurement was recorded shortly after adding 20 mM EDTA to the sample in order to remove all zinc bound to the protein. Measurements were performed on a LS 55 Luminescence Spectrometer (PerkinElmer, Waltham, United States). The samples were excited at 280 nm and emission spectra were recorded from 300 nm to 440.5 nm with a step size of 0.5 nm. For each sample, 10 consecutive measurements were averaged.

## Data availability statement

The datasets presented in this study can be found in online repositories. The names of the repository/repositories and accession number(s) can be found at: https://www.ncbi.nlm.nih.gov/geo/, GSE228855. https://www.rcsb.org/, 8Q5B, https://bmrb.io/, 34844.

## Author contributions

DÜ: Conceptualization, Writing – original draft, Writing – review & editing, Formal analysis, Investigation, Visualization. DP: Formal analysis, Investigation, Methodology, Visualization, Writing – original draft, Writing – review & editing. AB: Conceptualization, Methodology, Supervision, Visualization, Writing – review & editing. LH: Formal analysis, Investigation, Methodology, Writing – review & editing. RG: Investigation, Methodology, Writing – review & editing. RB: Funding acquisition, Project administration, Supervision, Writing – review & editing. CS: Formal analysis, Writing – review & editing, Funding acquisition, Supervision. HS: Formal analysis, Funding acquisition, Supervision, Writing – original draft, Writing – review & editing. JS: Funding acquisition, Supervision, Writing – review & editing, Conceptualization, Project administration, Writing – original draft.

## Footnotes

^1^http://www.ncbi.nlm.nih.gov/geo

^2^https://www.spp2002.uni-kiel.de/wordpress/

^3^www.halolex.mpg.de

^4^https://protocolexchange.researchsquare.com/article/pex-535/v1

^5^https://www.bioinformatics.babraham.ac.uk/projects/fastqc/
