# Supplementary material for: Characterization of the zinc finger μ-protein HVO_0758 from Haloferax volcanii: biological roles, zinc binding, and NMR solution structure
Source: Front Microbiol. 2023 Nov 29;14:1280972. doi: 10.3389/fmicb.2023.1280972 (PMC10716476; doi:10.3389/fmicb.2023.1280972)
Supplement: Supplementary file 1 [file Image_1.pdf]

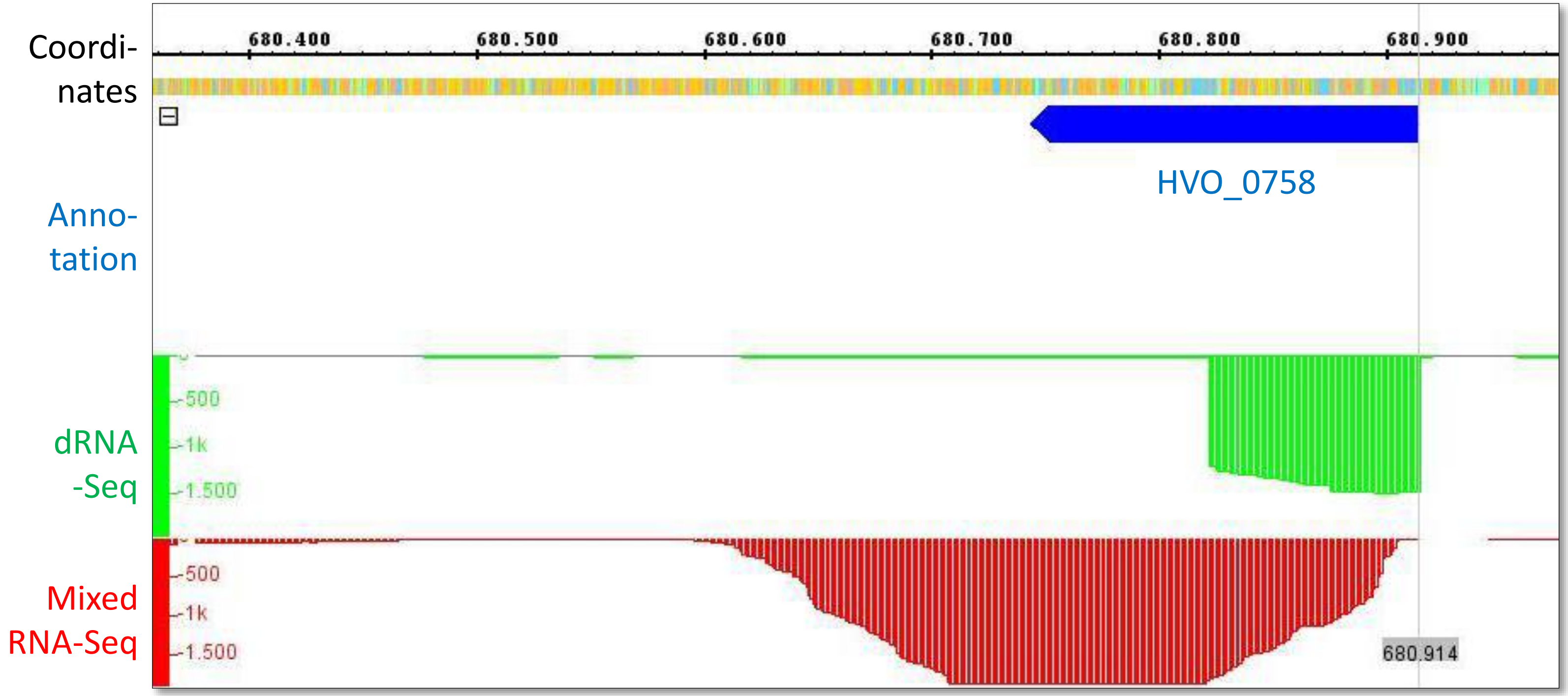

Supplementary Figure S1: Screenshot from the Integrated Genome Browser, showing the *HVO_0758* transcript. Results of a mixed RNA-Seq study shown in red (Laass et al., 2019) and a dRNA-Seq study shown in green (Babski et al., 2016) are presented together with the annotation shown in blue.
