# Supplementary material for: Characterization of the zinc finger μ-protein HVO_0758 from Haloferax volcanii: biological roles, zinc binding, and NMR solution structure
Source: Front Microbiol. 2023 Nov 29;14:1280972. doi: 10.3389/fmicb.2023.1280972 (PMC10716476; doi:10.3389/fmicb.2023.1280972)
Supplement: Supplementary file 2 [file Image_2.pdf]

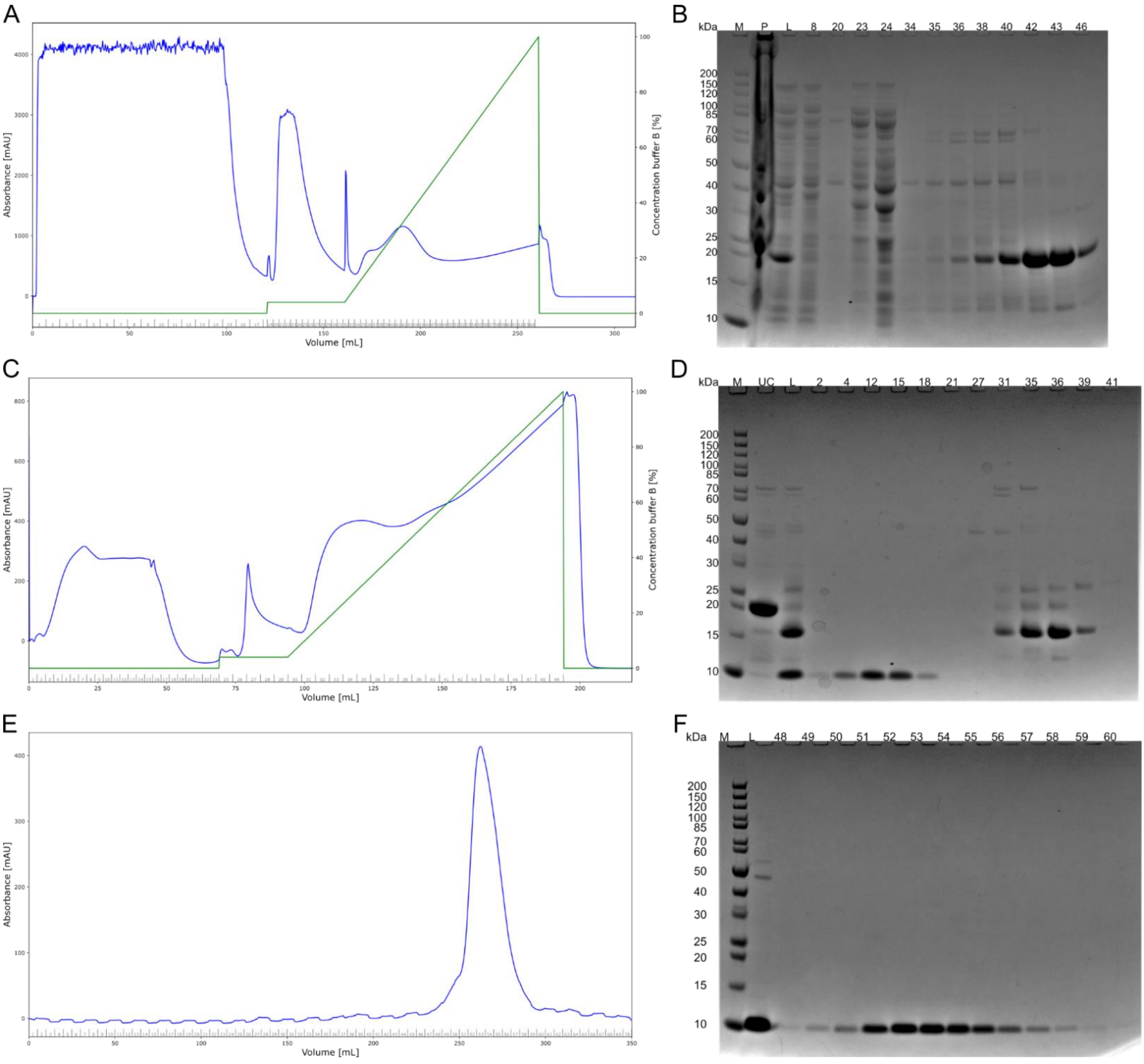

Supplementary Figure S2: Heterologous production and purification of HVO_0758. (A, C, E) Tandem metal affinity chromatography purification of HVO_0758 and final purification by size exclusion chromatography chromatography. The absorption at 280 nm is shown in blue. The elution gradients are shown in green. (B, D, F) The corresponding SDS-PAGE analyses of the relevant fractions from the chromatographies are shown.
