# Supplementary material for: Characterization of the zinc finger μ-protein HVO_0758 from Haloferax volcanii: biological roles, zinc binding, and NMR solution structure
Source: Front Microbiol. 2023 Nov 29;14:1280972. doi: 10.3389/fmicb.2023.1280972 (PMC10716476; doi:10.3389/fmicb.2023.1280972)
Supplement: Supplementary file 3 [file Image_3.pdf]

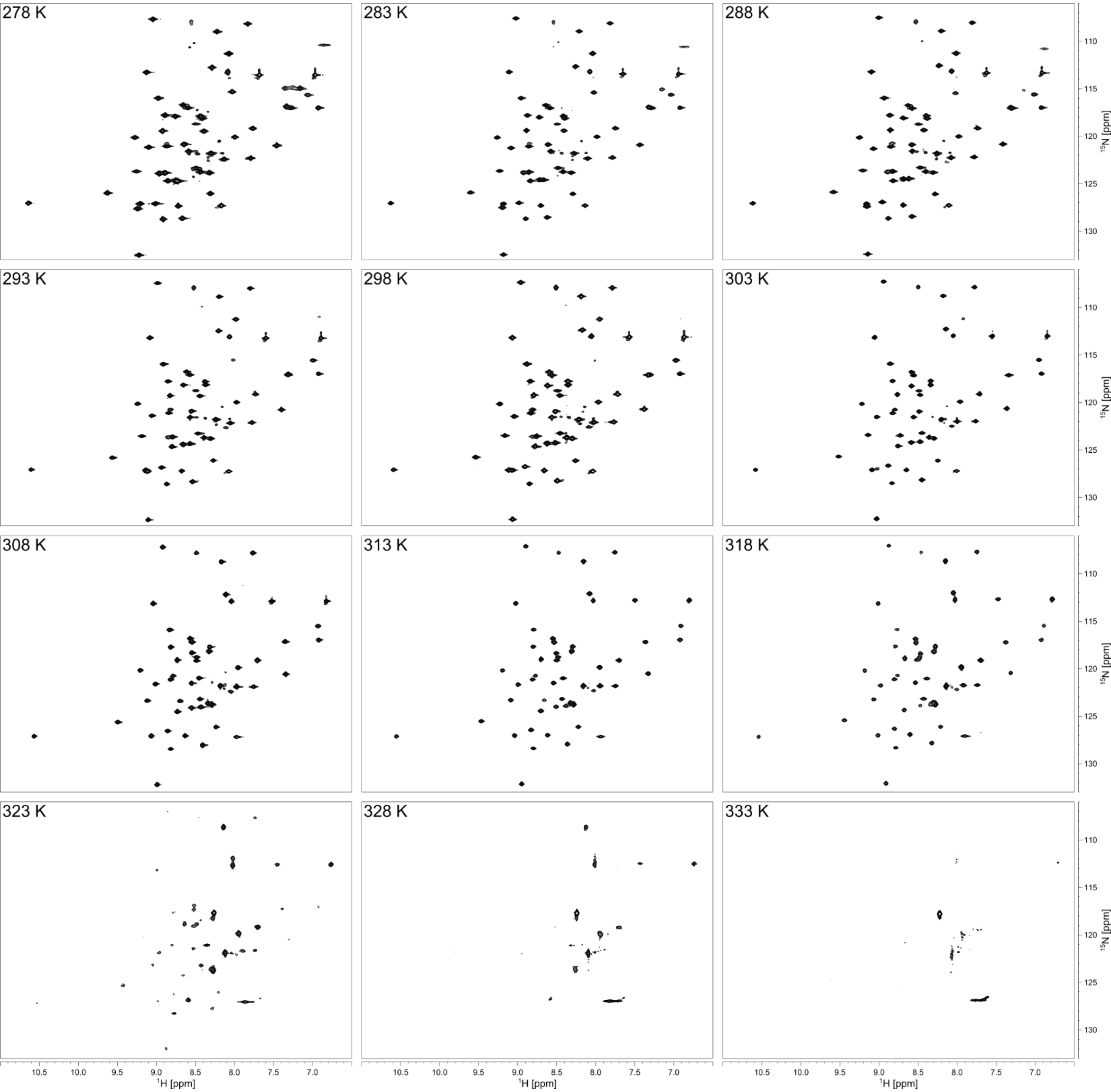

Supplementary Figure S3: Temperature dependence of NMR spectra: single spectra. 2D ^{1}H ^{15}N HSQC spectra of HVO_0758. The spectra were measured in temperature increments of 5K starting at 278 K up to 333 K. At 323 K, the protein visibly loses its structure, as signals start to vanish.
