# Supplementary material for: Characterization of the zinc finger μ-protein HVO_0758 from Haloferax volcanii: biological roles, zinc binding, and NMR solution structure
Source: Front Microbiol. 2023 Nov 29;14:1280972. doi: 10.3389/fmicb.2023.1280972 (PMC10716476; doi:10.3389/fmicb.2023.1280972)
Supplement: Supplementary file 4 [file Image_4.pdf]

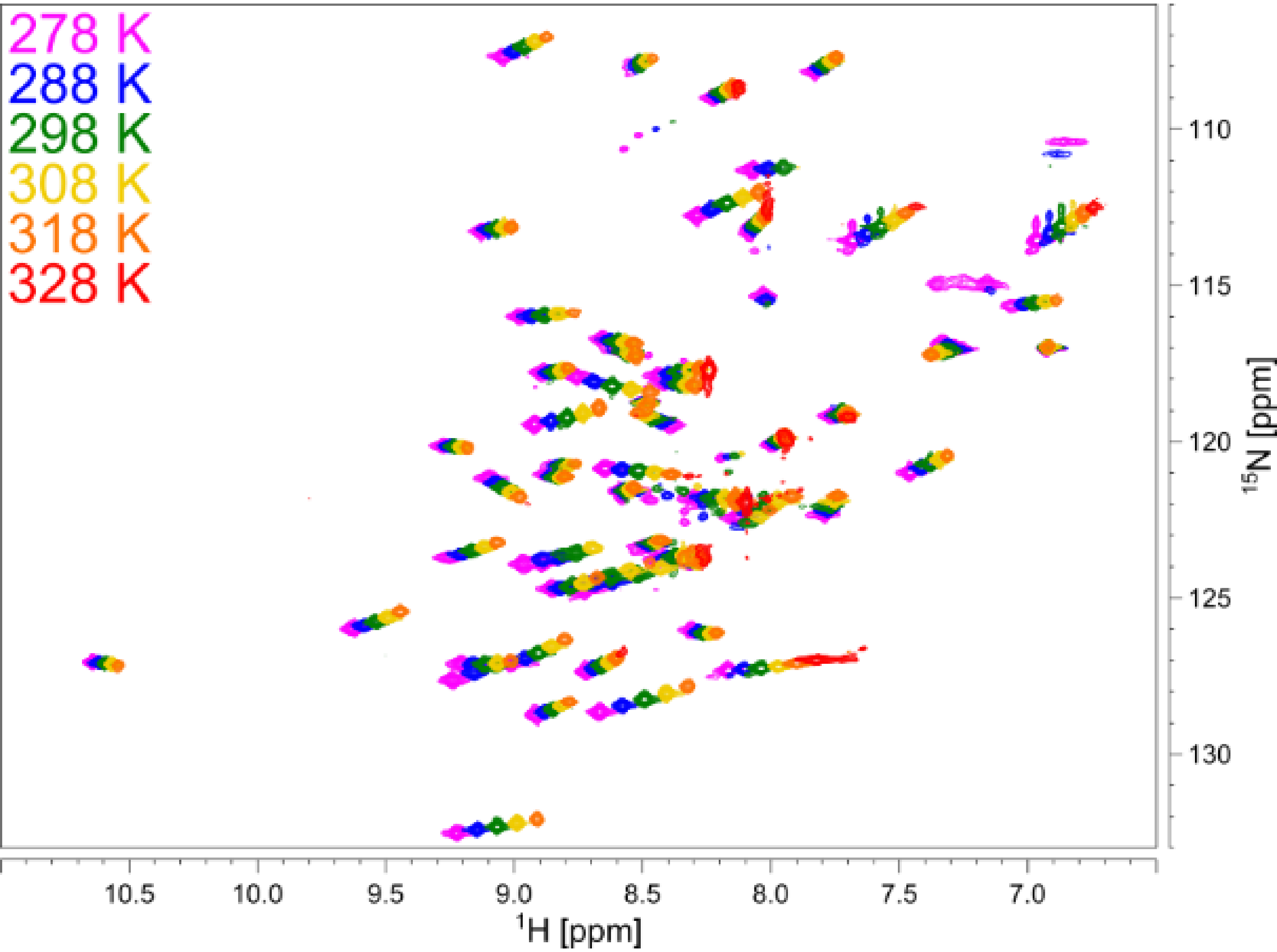

Supplementary Figure S4: Temperature dependence of NMR spectra: overlay. Overlay of 2D ^{1}H ^{15}N HSQC spectra of HVO_0758 at 278, 288, 298, 308, 318 and 328 K showing the various shifts of signals, which were used to calculate the temperature coefficients.
