# Supplementary material for: Characterization of the zinc finger μ-protein HVO_0758 from Haloferax volcanii: biological roles, zinc binding, and NMR solution structure
Source: Front Microbiol. 2023 Nov 29;14:1280972. doi: 10.3389/fmicb.2023.1280972 (PMC10716476; doi:10.3389/fmicb.2023.1280972)
Supplement: Supplementary file 5 [file Image_5.pdf]

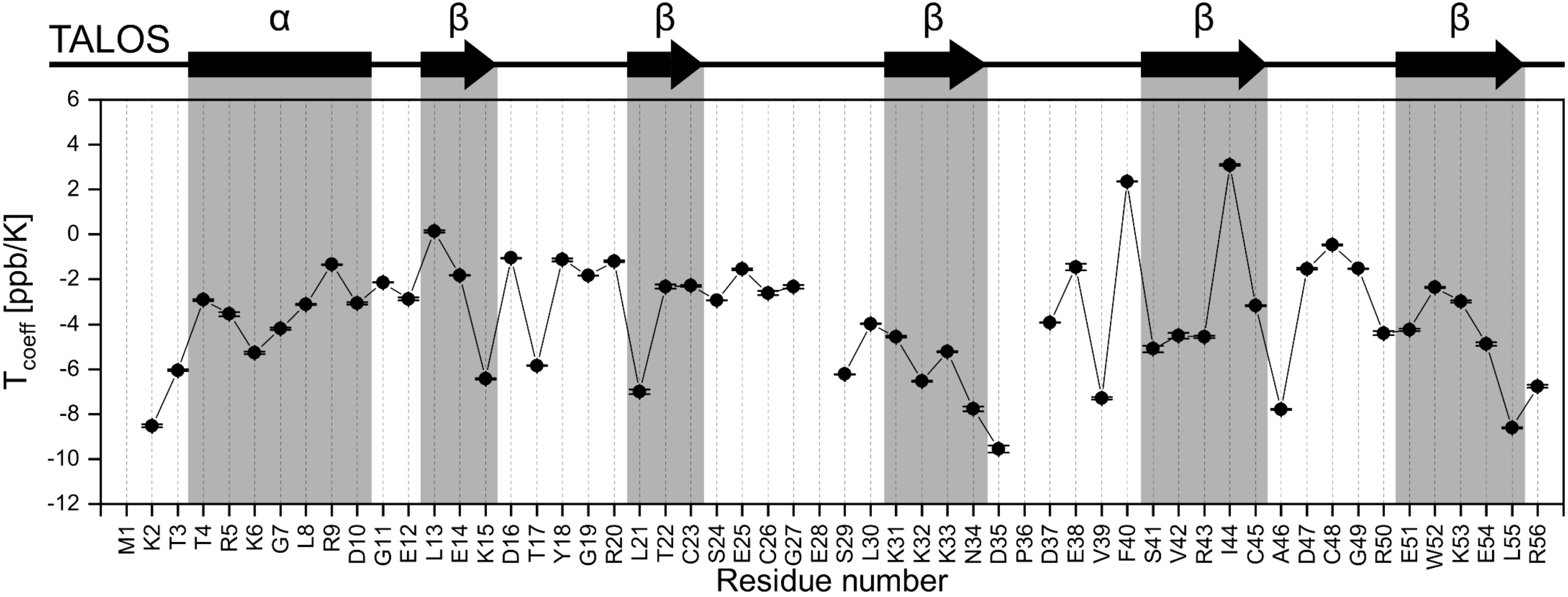

Supplementary Figure S5: Temperature coefficients. Calculated temperature coefficients T_{coeff} for HVO_0758. Values above -4.5 ppb/K indicate amide protons that are involved in hydrogen bonds, while values below -4.5 ppb/K indicate free amide protons.
