# Supplementary material for: Characterization of the zinc finger μ-protein HVO_0758 from Haloferax volcanii: biological roles, zinc binding, and NMR solution structure
Source: Front Microbiol. 2023 Nov 29;14:1280972. doi: 10.3389/fmicb.2023.1280972 (PMC10716476; doi:10.3389/fmicb.2023.1280972)
Supplement: Supplementary file 6 [file Image_6.pdf]

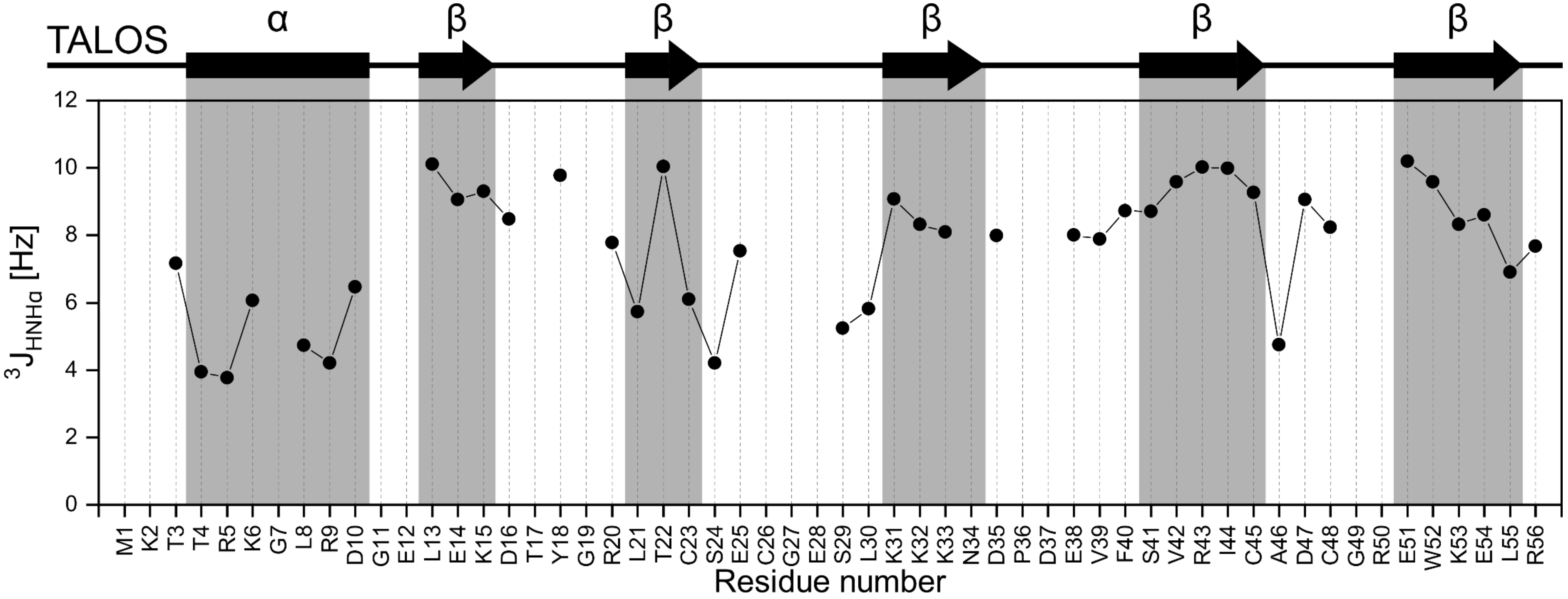

Supplementary Figure S6: $^3J_{HNH\alpha}$ coupling constants. $^3J_{HNHa}$ coupling constants for HVO_0758 calculated from a 3D HNHA spectrum. These can indicate the nature of secondary structure elements and be used in a structure calculation.
