# Supplementary material for: Characterization of the zinc finger μ-protein HVO_0758 from Haloferax volcanii: biological roles, zinc binding, and NMR solution structure
Source: Front Microbiol. 2023 Nov 29;14:1280972. doi: 10.3389/fmicb.2023.1280972 (PMC10716476; doi:10.3389/fmicb.2023.1280972)
Supplement: Supplementary file 7 [file Image_7.pdf]

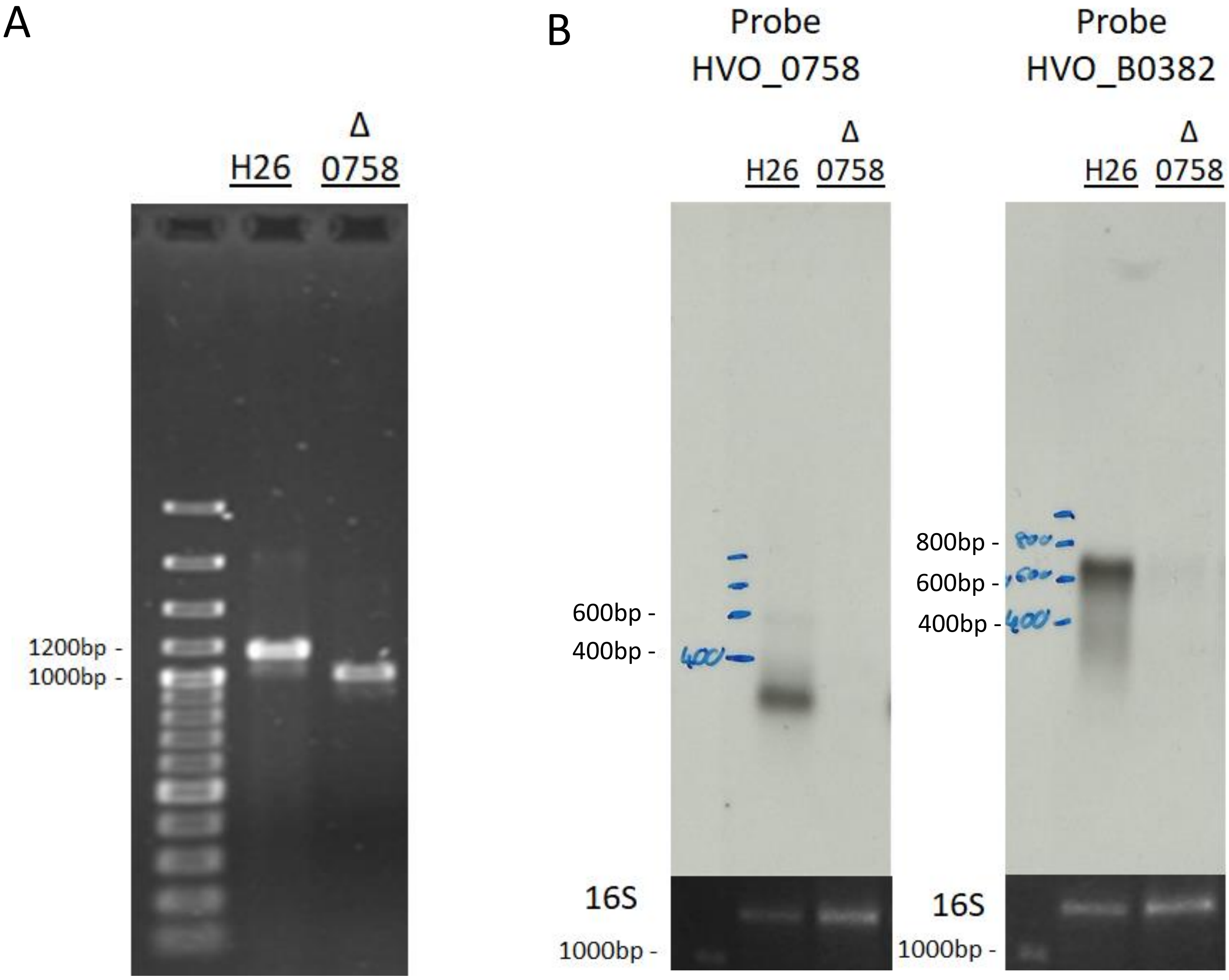

Supplementary Figure S7: Verification of the homozygocity of the deletion mutant by a multiple cycle PCR and the absence of the transcript by Northern blot analysis. A) PCR (40 cycles) for the verification of a homozygous deletion of the *HVO_0758* gene. Isolated genomic DNA of the H26 wild type and the *HVO_0758* in-frame deletion mutant (Δ0758; lacking a part of 147 nt within the ORF of *HVO_0758*) was analyzed with multicycle PCR using primers (0758_P1 & 0758_P4; see Supplementary Table S4) flanking the *HVO_0758* genomic locus. Calculated PCR product length for the HVO_0758 wild type locus is 1165bp and for the deletion 1018bp. The deletion was checked frequently via PCR during experimental analyses using the ΔHVO_0758 strain.
B) Full length Northern blot analyses of the expression of the genes *HVO_0758* (left; transcript ca. 300nt) and *HVO_B0382* (right; transcript ca. 650nt) in the wild-type (H26) and *HVO_0758* deletion mutant (Δ0758). 16S rRNA served as loading control/reference.
