# Supplementary material for: Characterization of the zinc finger μ-protein HVO_0758 from Haloferax volcanii: biological roles, zinc binding, and NMR solution structure
Source: Front Microbiol. 2023 Nov 29;14:1280972. doi: 10.3389/fmicb.2023.1280972 (PMC10716476; doi:10.3389/fmicb.2023.1280972)
Supplement: Supplementary file 8 [file Image_8.pdf]

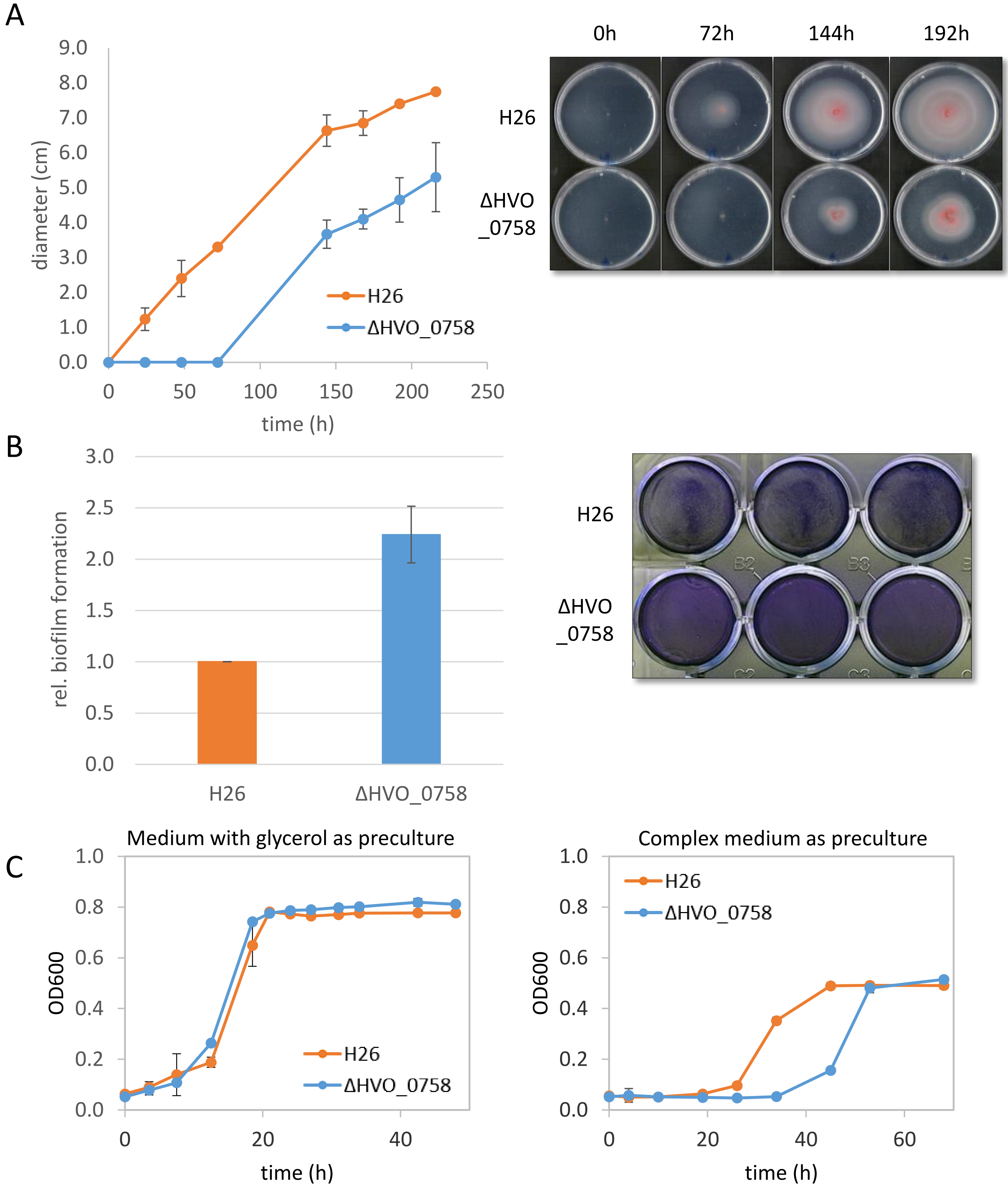

Supplementary Figure S8: Phenotypical characterization of the *HVO_0758* deletion mutant (ΔHVO_0758) in comparison with the H26 wild-type (WT/H26). (A) Swarming assay. The swarming assay was performed in petri dishes (diameter 9cm). Left: Data shown of three biological replicates (mean values and SD). Right: exemplary pictures of dishes showing the swarming progression at the indicated time points. (B) Biofilm assay. The biofilm assay was performed in 24-well plates. Left: mean values and SD of relative biofilm formation (n≥3) normalized to wild type. Right: Exemplary pictures of 24-wells after crystal violet staining. (C) Growth curves of both strains in synthetic medium with glycerol as carbon source. Left: Synthetic medium with glycerol as carbon source as preculture. Right: Complex medium as preculture. Growth analyses were performed in round-bottom 96-well plates. The OD600 was measured at the given time points and mean values and SD of three biological replicates are shown.
