# Supplementary material for: Characterization of the zinc finger μ-protein HVO_0758 from Haloferax volcanii: biological roles, zinc binding, and NMR solution structure
Source: Front Microbiol. 2023 Nov 29;14:1280972. doi: 10.3389/fmicb.2023.1280972 (PMC10716476; doi:10.3389/fmicb.2023.1280972)
Supplement: Supplementary file 9 [file Image_9.pdf]

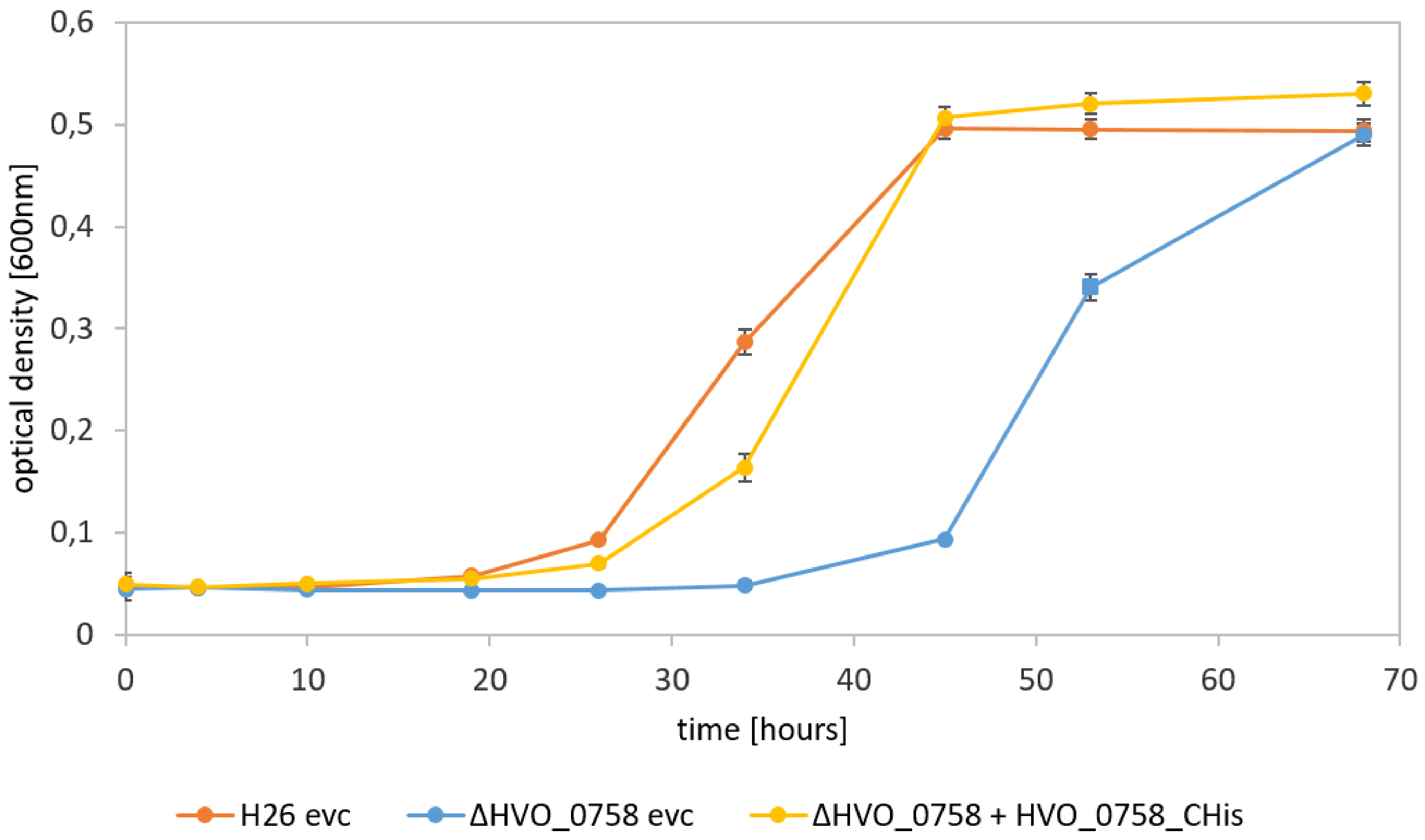

Supplementary Figure S9: Complementation of the *HVO_0758* deletion mutant growth phenotype in glycerol medium. Presented are growth curves of the parent strain H26 with the empty vector control (H26 evc) in orange, the deletion mutant ΔHVO_0758 with the empty vector (ΔHVO_0758 evc) in blue and the complementation strain ΔHVO_0758+HVO_0758CHis in yellow. Complex medium was used for preculture before shifting to synthetic medium with glycerol as carbon source. Mean values and SD of six technical replicates are shown. One representative of three biological replicates is presented.
