# Supplementary material for: Characterization of the zinc finger μ-protein HVO_0758 from Haloferax volcanii: biological roles, zinc binding, and NMR solution structure
Source: Front Microbiol. 2023 Nov 29;14:1280972. doi: 10.3389/fmicb.2023.1280972 (PMC10716476; doi:10.3389/fmicb.2023.1280972)
Supplement: Supplementary file 10 [file Image_10.pdf]

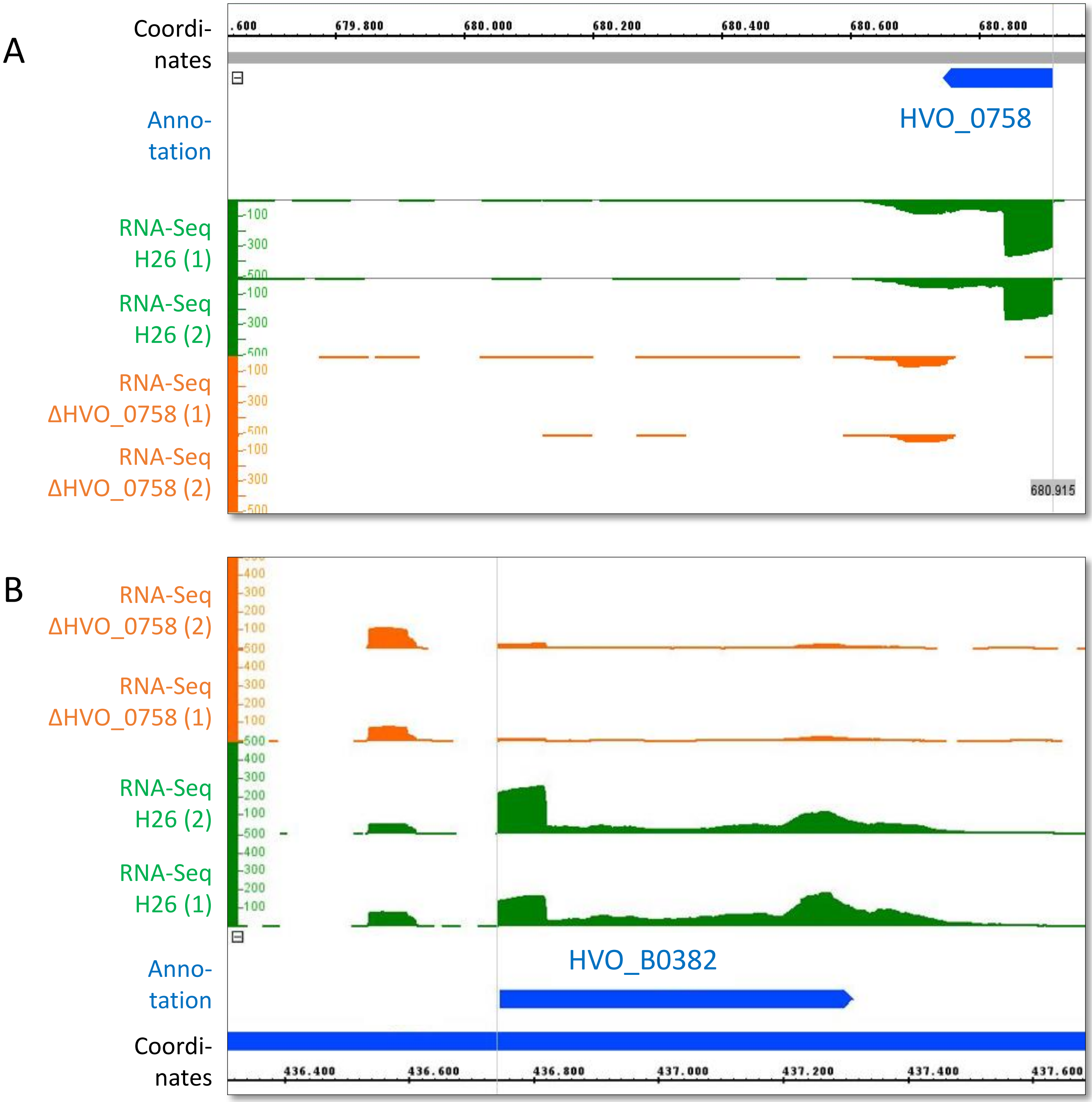

Supplementary Figure S10: RNA-Seq results of the transcript levels of *HVO_0758* (A) and *HVO_B0382* (B) in the H26 wild-type (H26 shown in green) and the *HVO_0758* deletion mutant (ΔHVO_0758, shown in orange). Reads from two biological replicates are depicted, respectively. Screenshots were taken from the Integrated Genome Browser.
