# Supplementary material for: Characterization of the zinc finger μ-protein HVO_0758 from Haloferax volcanii: biological roles, zinc binding, and NMR solution structure
Source: Front Microbiol. 2023 Nov 29;14:1280972. doi: 10.3389/fmicb.2023.1280972 (PMC10716476; doi:10.3389/fmicb.2023.1280972)
Supplement: Supplementary file 11 [file Image_11.pdf]

A

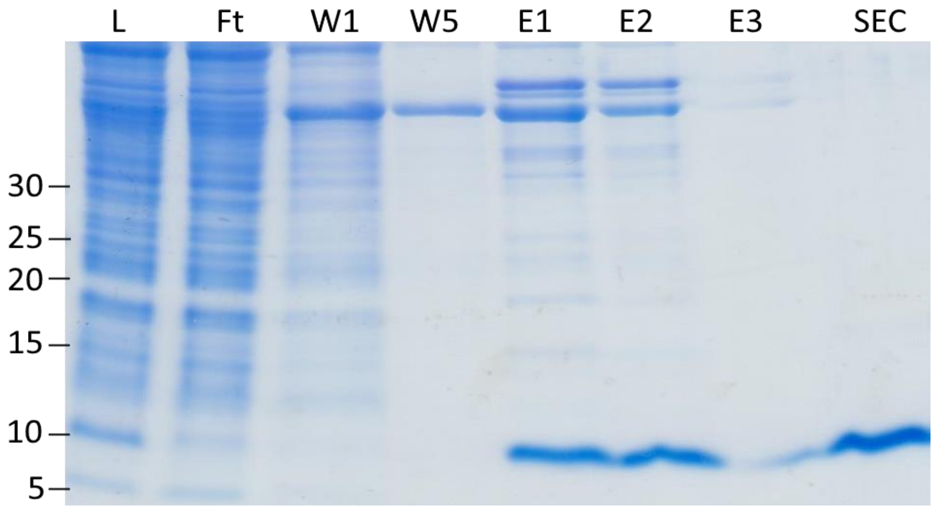

B

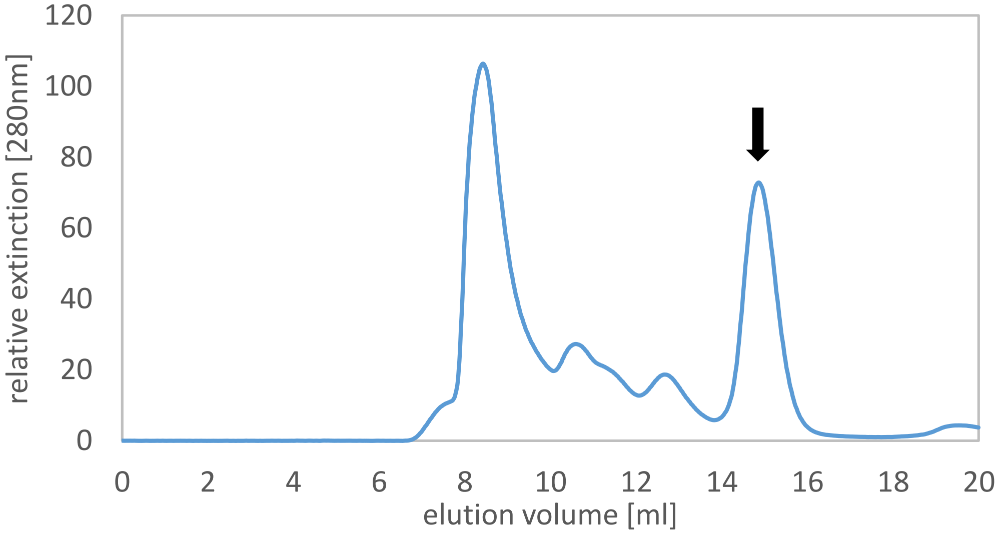

Supplementary Figure S11: Homologous production and purification of HVO_0758. Two-step purification of homologously overproduced HVO_0758_CHis. (A) Tricine-SDS-PAGE analysis of relevant fractions from the nickel affinity chromatography and subsequent size exclusion chromatography (kDa range indicated on the left; his-tagged HVO_0758 ≈ 7.5 kDa) (B) Chromatogram of the SEC of the IMAC elution fraction of HVO_0758_CHis. The arrow indicates the peak corresponding to the target protein.
