# Supplementary material for: Characterization of the zinc finger μ-protein HVO_0758 from Haloferax volcanii: biological roles, zinc binding, and NMR solution structure
Source: Front Microbiol. 2023 Nov 29;14:1280972. doi: 10.3389/fmicb.2023.1280972 (PMC10716476; doi:10.3389/fmicb.2023.1280972)
Supplement: Supplementary file 12 [file Image_12.pdf]

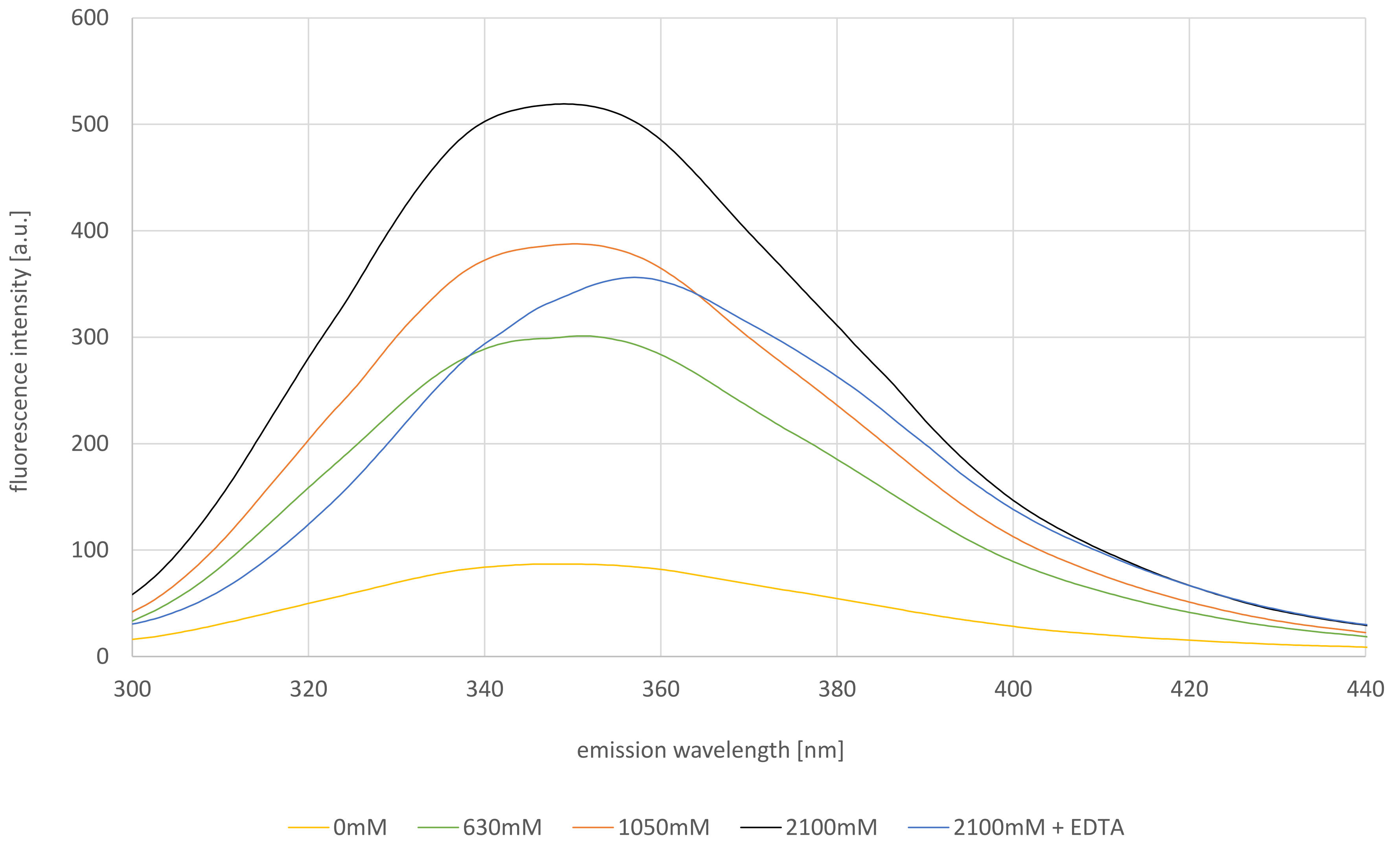

Supplementary Figure S12: Raw fluorescence emission spectra of HVO_0758 in buffers containing different NaCl concentrations, as indicated below. The complete wavelength range from 300 nm to 440 nm is shown. Ten consecutive measurements per sample were performed and averaged.
