# Supplementary material for: Characterization of the zinc finger μ-protein HVO_0758 from Haloferax volcanii: biological roles, zinc binding, and NMR solution structure
Source: Front Microbiol. 2023 Nov 29;14:1280972. doi: 10.3389/fmicb.2023.1280972 (PMC10716476; doi:10.3389/fmicb.2023.1280972)
Supplement: Supplementary file 13 [file Image_13.pdf]

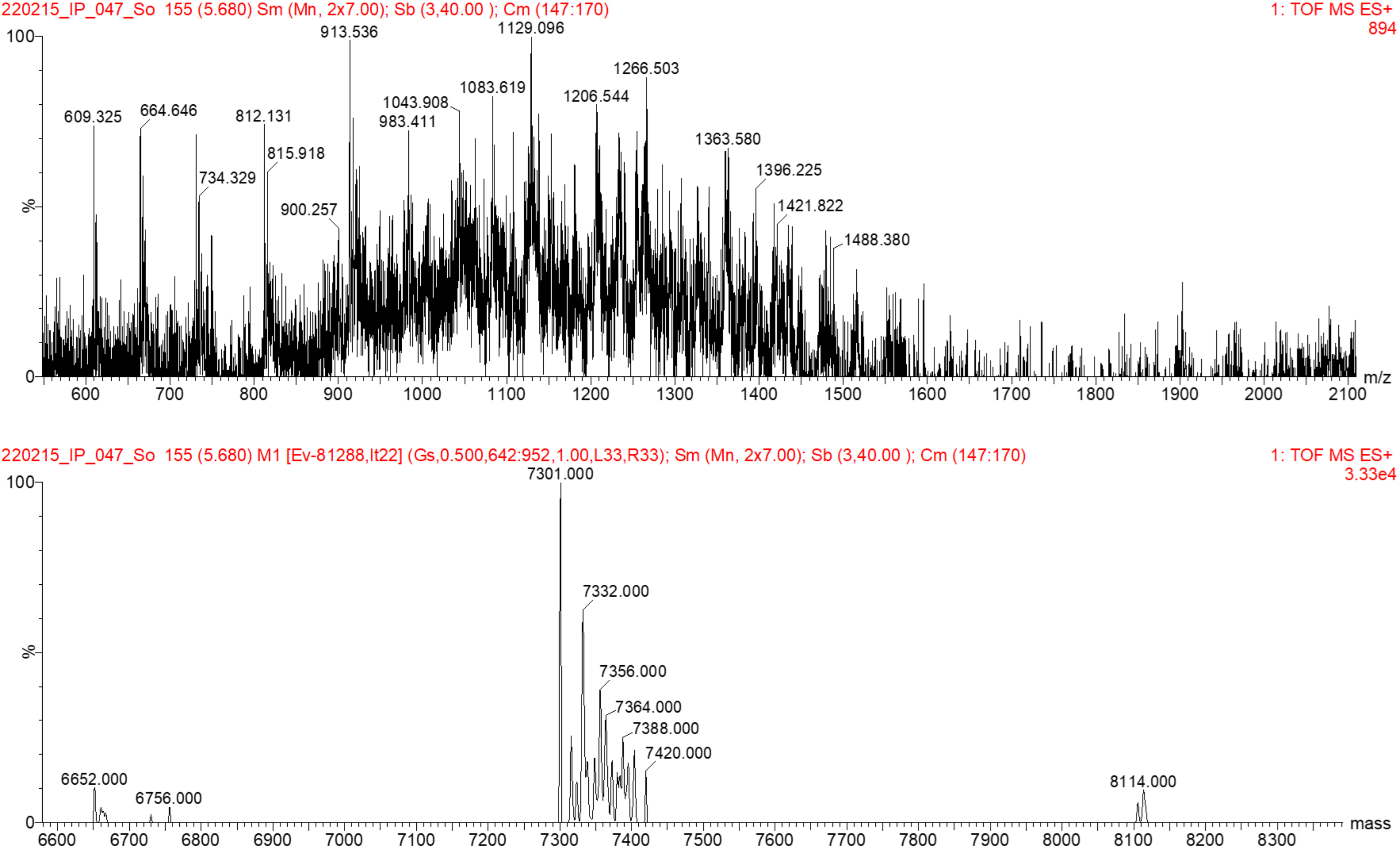

Supplementary Figure S13: Mass spectrum of HVO_0758. Positive electrospray ionisation time-of-flight mass spectrum of HVO_0758 (intact protein measurement). The calculated masses and their relative abundances are shown on the bottom.
