# Supplementary material for: Characterization of the zinc finger μ-protein HVO_0758 from Haloferax volcanii: biological roles, zinc binding, and NMR solution structure
Source: Front Microbiol. 2023 Nov 29;14:1280972. doi: 10.3389/fmicb.2023.1280972 (PMC10716476; doi:10.3389/fmicb.2023.1280972)
Supplement: Supplementary file 14 [file Image_14.pdf]

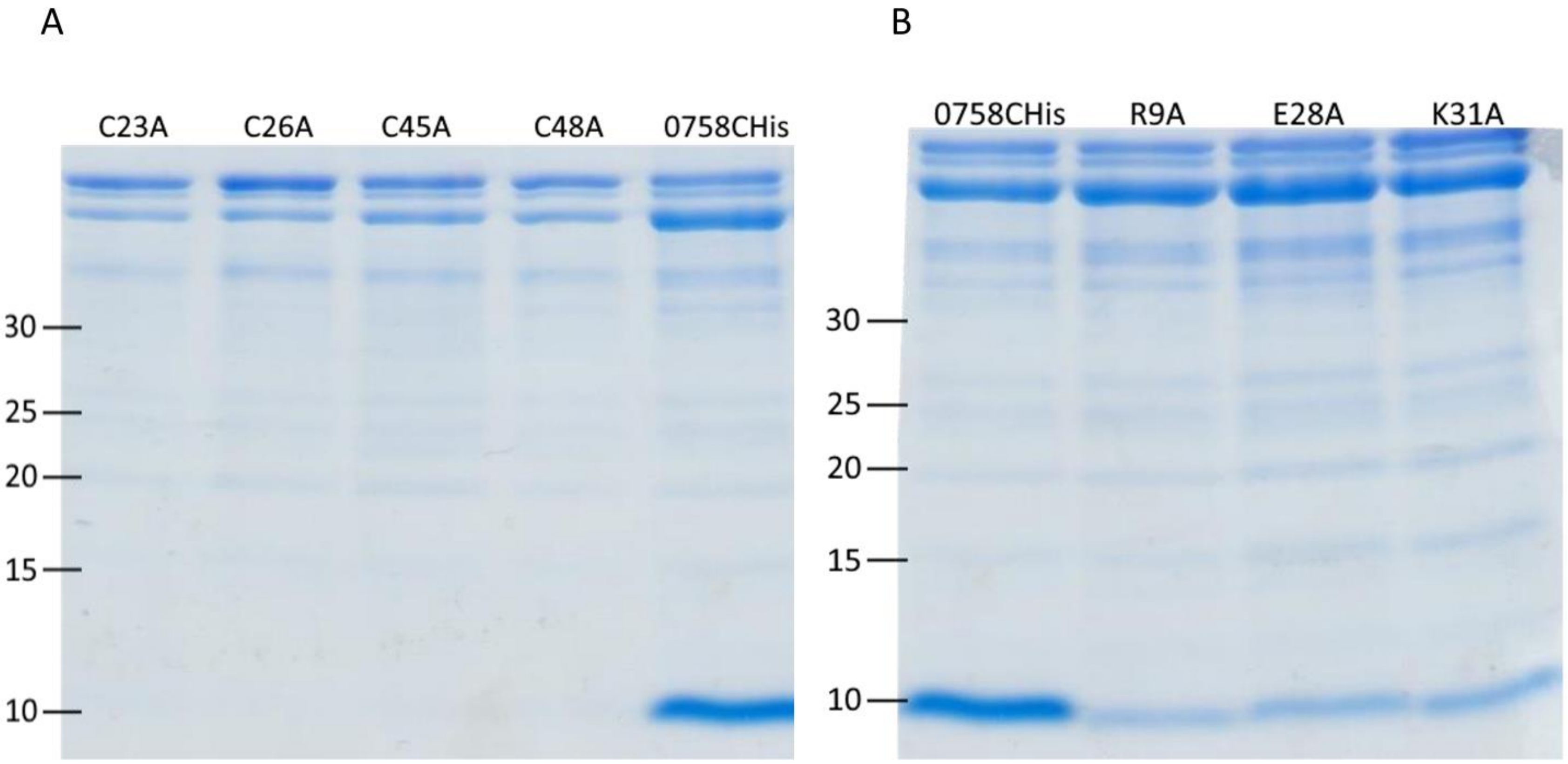

Supplementary Figure S14: Tricine-SDS-PAGE analyses of nickel chelating chromatography elution fractions of the C-terminal Hexahistidin tagged wild-type HVO_0758 and several point mutants, as indicated at the top. **A.** Results for four cysteine -> alanine point mutants. **B.** Results for three additional point mutants. The sizes of molecular weight marker bands are shown to the left of the gels.
