# Supplementary material for: Characterization of the zinc finger μ-protein HVO_0758 from Haloferax volcanii: biological roles, zinc binding, and NMR solution structure
Source: Front Microbiol. 2023 Nov 29;14:1280972. doi: 10.3389/fmicb.2023.1280972 (PMC10716476; doi:10.3389/fmicb.2023.1280972)
Supplement: Supplementary file 17 [file Table_3.docx]

**Supplementary Table S3.** Chemotaxis gene Cluster in *H. volanii*. Genes that are downregulated at least twofold in the deletion mutants ΔHVO_0578 and ΔHVO_2753 are indicated. Gene names and annotated functions are also tabulated.

| Gene ID  HVO_ | Down in  ΔHVO_0758 | Down in  ΔHVO_2753 | Gene Name | Annotated Function |
| --- | --- | --- | --- | --- |
| 1201 | X | X |  | sensor box histidine kinase |
| 1202 | X | X |  | conserved hypothetical protein |
| 1203 | X | X | arlD1 | arl cluster protein ArlD |
| 1204 | X | X |  | non-functional |
| 1205 | X | X | cheD | taxis cluster protein CheD |
| 1206 | X | X | cheC | taxis cluster protein CheC |
| 1207 |  | X | cheY | response regulator CheY |
| 1208 |  | X |  | conserved hypothetical protein |
| 1209 |  | X |  | HTH domain protein |
| 1210 | X | X | arlA1 | archaellin A1 |
| 1211 | X | X | arlA2 | archaellin A2 |
| 1212 |  | X | cirA | KaiC-type circadian clock protein |
| 1213 | X | X | arlCE | arl cluster protein ArlCE |
| 1214 | X | X | arlF | arl cluster protein ArlF |
| 1215 |  | X | arlG | arl cluster protein ArlG |
| 1216 |  | X | arlH | arl cluster protein ArlH |
| 1217 |  | X | arlI | archaellar motor/biogenesis |
| 1218 |  | X | arlJ | archaellar motor/biogenesis |
| 1219 |  | X | cheF2 | archaellar motor/biogenesis |
| 1220 |  | X |  | conserved hypothetical protein |
| 1221 |  | X | cheF1 | taxis protein CheF1 |
| 1222 |  | X | cheR | methyltransferase CheR |
| 1223 |  | X | cheA | taxis sensor histidine kinase |
| 1224 |  | X | cheG | protein-glutamate methylesterase |
| 1225 | X | X | cheW1 | purine-binding taxis protein |
